# Electrospun PVA Fibers for Drug Delivery: A Review

**DOI:** 10.3390/polym15183837

**Published:** 2023-09-20

**Authors:** Fatima T. Zahra, Quincy Quick, Richard Mu

**Affiliations:** 1 TIGER Institute, Tennessee State University, Nashville, TN 37209, USA; 2 Department of Biological Sciences, Tennessee State University, Nashville, TN 37209, USA

**Keywords:** electrospinning, polyvinyl alcohol (PVA), nanofibers, drug delivery, wound dressing, transdermal, tissue engineering

## Abstract

Innovation in biomedical science is always a field of interest for researchers. Drug delivery, being one of the key areas of biomedical science, has gained considerable significance. The utilization of simple yet effective techniques such as electrospinning has undergone significant development in the field of drug delivery. Various polymers such as PEG (polyethylene glycol), PLGA (Poly(lactic-co-glycolic acid)), PLA(Polylactic acid), and PCA (poly(methacrylate citric acid)) have been utilized to prepare electrospinning-based drug delivery systems (DDSs). Polyvinyl alcohol (PVA) has recently gained attention because of its biocompatibility, biodegradability, non-toxicity, and ideal mechanical properties as these are the key factors in developing DDSs. Moreover, it has shown promising results in developing DDSs individually and when combined with natural and synthetic polymers such as chitosan and polycaprolactone (PCL). Considering the outstanding properties of PVA, the aim of this review paper was therefore to summarize these recent advances by highlighting the potential of electrospun PVA for drug delivery systems.

## 1. Introduction

**Figure 1:**
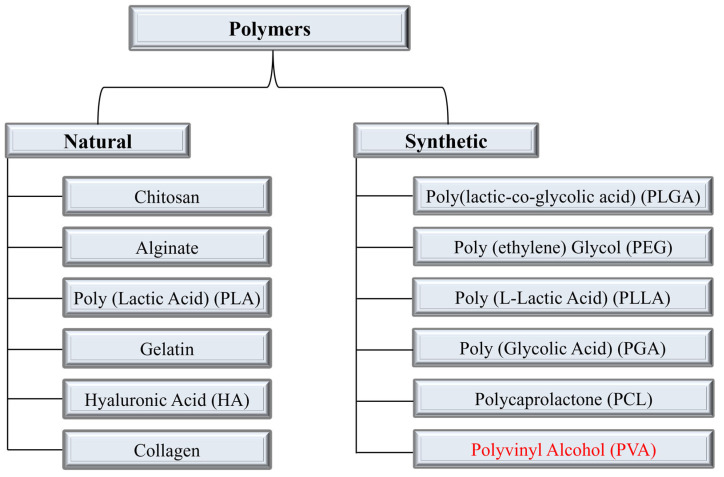
Categories of polymers and the most used polymers in each category for DDSs. The polymer under discussion Polyvinyl Alcohol (PVA) is highlighted in synthetic polymers category.

The term “drug delivery” refers to administrating the therapeutic effect of a pharmaceutical compound to humans or animals. Progress in the field of disease exploration is widely acknowledged, leading to ongoing research and development of advanced techniques for advanced drug delivery systems (DDSs). Some of the key factors in developing an effective and controlled drug delivery system include its inertness, non-toxicity, biocompatibility, high mechanical strength, patient comfort, and high drug-loading capability. To streamline and optimize the performance of this domain, an extensive array of polymers has been researched and effectively employed. The polymeric drug delivery systems have various categories such as microparticles, micelles, hydrogels/transplants, nanoparticles, and drug conjugates [1,2,3,4,5,6,7]. These systems are employed to improve controlled drug release, targeted drug delivery, and solubility. Generally, polymers are categorized as natural or synthetic polymers. Figure 1 illustrates the most frequently used polymers in both categories for the development of DDSs. Natural polymers are sometimes blended with synthetic compounds to chemically modify their functional groups, resulting in what is known as semi-synthetic polymers. Chitosan (CS) is one of the most commonly explored natural polymers and various studies have been reported on its potential in developing advanced drug delivery systems [8,9,10,11,12]. Synthetic polymers are sub-divided into hydrophobic and hydrophilic groups which are utilized in DDSs as core–shell nanoparticles depending on the required criteria. The drug release and drug loading efficiency of hydrophilic polymers is believed to be higher than those of hydrophobic polymers [13]. Combining hydrophobic and hydrophilic synthetic polymers in various proportions has also been employed for the development of drug nanocarriers. For instance, the combination of poly(ethylene glycol) (PEG) and polycaprolactone (PCL) has been utilized to investigate the endocytosis pathways into cells [14]. Polymeric capsules have been developed for cancer therapy by incorporating targeting ligands, thereby mitigating the accompanying side effects and toxicity [15]. Polymers are commonly used to develop transdermal DDSs, wound dressings, cancer therapy, tissue engineering, and oral DDSs. The disadvantages associated with commonly used synthetic/natural polymers such as PCL, PLGA (poly(lactic-co-glycolic acid)), and PLA (poly(lactic acid)) include the slow degradation and biocompatibility issues due to the formation of acidic degradation products [16,17].

**Figure 2:**
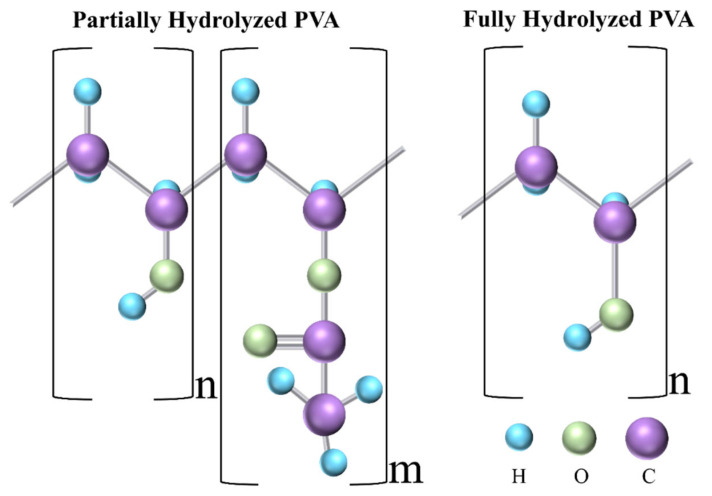
Molecular structure of partially and fully hydrolyzed PVA. C, O, and H refer to carbon, oxygen, and hydrogen, respectively.

Polyvinyl alcohol (PVA) is a synthetic biopolymer that has been used for decades as a blending polymer with natural and synthetic polymers to improve the efficacy of DDSs. It is synthesized by the hydrolysis of polyvinyl acetate (PVAc) [18]. Depending on the degree of hydrolysis, which varies between 80 and 99%, PVA [CH_2_CH(OH)]_n_ is subdivided into two categories, i.e., partially hydrolyzed and fully hydrolyzed. Figure 2 shows the molecular structure of both categories. 

PVA possesses a semicrystalline solid structure and is not only biocompatible, biodegradable, non-toxic, and hydrophilic, but also has a good spinning capability, and thus holds significant potential in electrospinning-based DDSs. There have been a few review papers that have examined the use of PVA in drug delivery systems for specific applications, such as tissue engineering and cancer therapy [19,20]. However, to the best of our knowledge, there is currently a lack of a comprehensive review that specifically examines the potential of drug delivery systems based on the electrospinning of PVA. Therefore, this review aims to fill this gap by providing a comprehensive analysis of the existing literature, highlighting the significance of utilizing electrospun PVA drug delivery systems.

## 2. Electrospinning for DDSs

**Figure 3:**
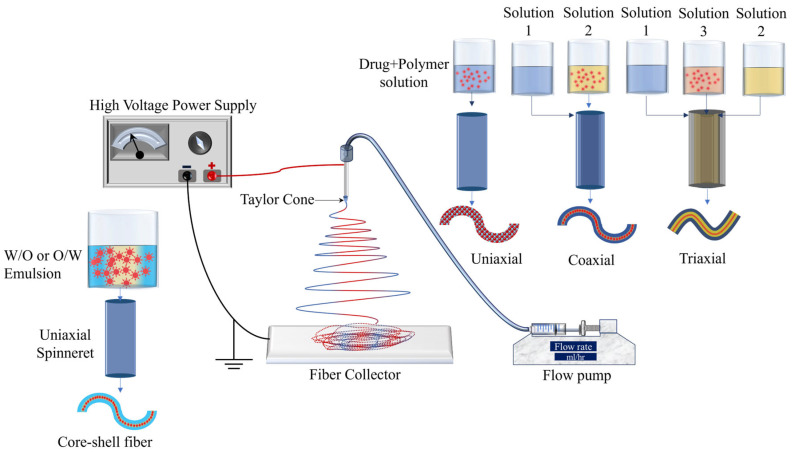
Schematic of electrospinning and its types.

Electrospinning is a cost-effective and simple tool that is used for the preparation of drug delivery systems using natural, synthetic, and blended polymers. Figure 3 shows the schematic of the electrospinning process. In the electrospinning process, generally, the flow pump, spinneret, collector (e.g., a plane conducting sheet, rotating drum), and high voltage power supply are the main components. The spinneret is connected to a high-voltage source while the collector is grounded. The flow rate is set using the flow pump and it depends mainly on the viscosity of the polymer solution. Once the voltage is applied, the positively charged solution travels from the needle tip toward the grounded collector. The formation of the Taylor cone on the tip of the needle is an important factor without which the fibers may not be formed. Electrospinning has excessively been used for the development of different drug delivery systems and the process parameters mentioned above significantly affect the fiber morphology. For example, a higher flow rate can lead to high bead density, higher viscosity leads to an increase in diameter, lower viscosity results in bead formation, and longer needle-to-collector distance can result in instability of the electric field and hence negatively affect the fiber formation [21]. Therefore, it can be concluded that optimization of the processing parameters is an important phase in obtaining fine and bead-free fibers and surface morphology. Natural and synthetic polymers that have been most widely used in electrospun drug delivery systems are listed in Table 1. 

One of the primary benefits of utilizing electrospinning in drug delivery systems lies in its ability to regulate the rate of drug release. This control is achieved by manipulating the degradation rate of the fibers through factors such as molecular weight and distribution, porosity of the fibers, and composition. The volatility of the solvent is a crucial consideration, particularly in the development of drug delivery systems (DDSs), as it significantly influences the achievement of the desired porosity of the fibers. This porosity is a critical factor in obtaining the desired drug release characteristics. Non-volatile solvents result in the generation of fibers characterized by larger pore sizes and reduced surface area, which is unfavorable for drug delivery applications [41,42]. Alongside solution volatility, solution viscosity, surface tension, and conductivity are noteworthy attributes that impact the morphology of the fibers. The viscosity of the solution is directly influenced by the concentration of the polymer employed, and it serves as a crucial parameter for controlling the spinnability of the solution. Lower viscosity is associated with the production of thin fibers that exhibit favorable mechanical strength. However, when the viscosity of the solution is very low, specifically below 1 poise, it results in the formation of beads and has a detrimental impact on the spinnability of the solution. Similarly, the presence of a significantly high viscosity, exceeding 20 poise, impedes the flow of the polymer, thereby preventing the successful fabrication of fibers. It is widely believed that in order to achieve optimal fiber morphology, the recommended viscosity range is between 1 and 20 poise, with a corresponding surface tension range of 35–55 dyn/cm^2^ [43]. Additionally, the conductivity of the solution is a crucial parameter that primarily influences the formation of the Taylor cone. If the surface of the droplet does not possess sufficient charge, it can hinder the formation of the Taylor cone, thereby potentially impeding the process of fiber formation. Additionally, it has been observed that the diameter of the fibers decreases as the solution conductivity increases. This phenomenon can be attributed to the application of tensile force in the presence of an electric field [44,45,46]. 

As shown in Figure 3, uniaxial, coaxial, and triaxial electrospinning is utilized for the preparation of monolithic fibers, core–shell fibers, and triaxial fibers, respectively, for the development of DDSs. In the case of uniaxial fiber fabrication, a single spinneret is used with a solution containing a combination of a drug and a polymer. For core–shell fibers, two spinnerets are utilized, each with its own solution: one for the core (typically a drug) and another for the shell (a polymer). Lastly, three spinnerets are employed for triaxial fibers, with one for the shell (a polymer), another for an intermediate layer, and a third for the core material [21]. Encapsulation of drugs in the electrospun fibers has been utilized for the development of controlled-release therapeutic elements in a timely manner to avoid potential side effects [47]. Moreover, electrospun fiber scaffolds have also been utilized for the restoration as well as repair of tissues/organs [48]. 

Emulsion electrospinning (Figure 3) is a type of uniaxial (needle-less (rotating electrode) or with a needle) electrospinning technique that is used to prepare core–shell fibrous structures using water-in-oil (W/O) or oil-in-water (O/W) emulsions to encapsulate hydrophilic or hydrophobic compounds, respectively. The emulsion consists of two liquid phases: an aqueous phase (hydrophilic) and an oil phase (hydrophobic). The drug is dissolved in the aqueous phase and then dispersed in the organic polymer solution (oil phase). The oil phase, characterized by a higher evaporation rate, exhibits increased viscosity. During the emulsion electrospinning process, the aqueous phase, containing the drug, merges into the core of the fiber structure, while the oil phase tends to migrate towards the periphery. This technique has demonstrated efficacy in achieving prolonged drug release.

**Figure 4:**
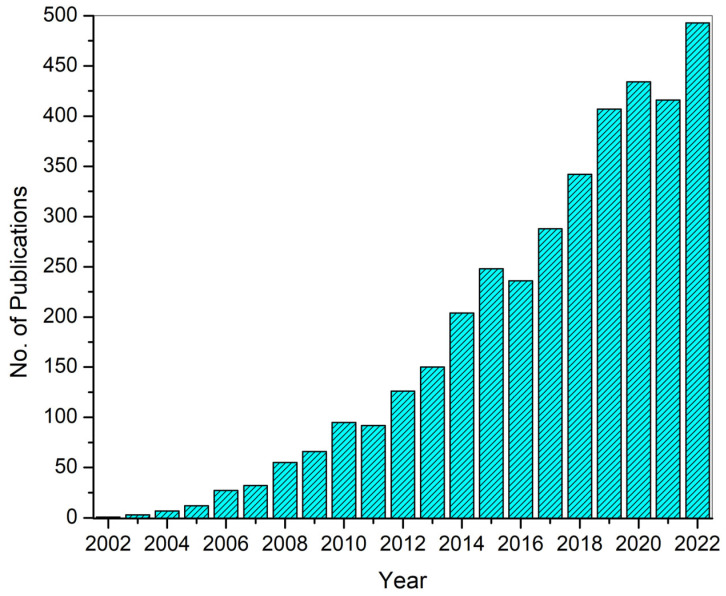
Number of publications until 2022 on electrospinning-based DDSs. The data were extracted from Web of Science (WoS).

Figure 4 shows the publication data until 2022 for keywords “electrospinning” and “drug delivery” extracted from Web of Science. More than 200 papers in the same field have already been published in 2023. This clearly shows the high potential of electrospinning and the interest of researchers in electrospinning to develop drug delivery systems.

## 3. Electrospinning-Based PVA DDSs

**Figure 5:**
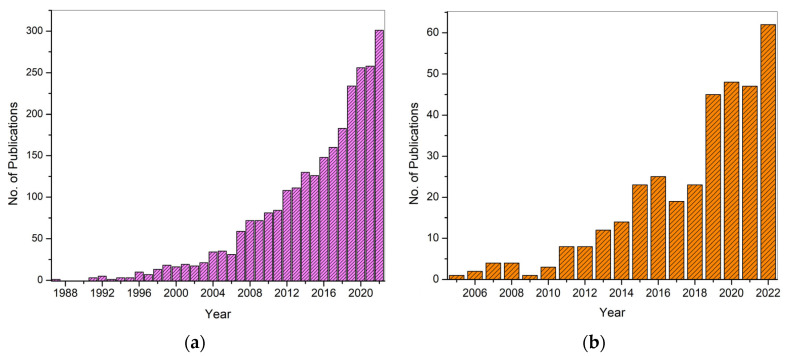
Number of publications until 2022 on PVA in drug delivery systems (**a**), and electrospinning-based PVA DDSs until 2022 (**b**).

As previously mentioned, PVA is generally classified into two main categories, i.e., partially hydrolyzed and fully hydrolyzed. The extent of hydrolysis has an impact on the physical characteristics of PVA, including its molecular weight (ranging from 20,000 to 400,000 g/mol), solubility, and mechanical properties. Consequently, the appropriate category of PVA is selected to meet the specific demands of a given drug delivery system. The hydrophilic nature, non-toxicity, and high mechanical strength of PVA render it an optimal material for use in polymeric drug delivery systems. It should be noted that polyvinyl alcohol (PVA) is commonly crosslinked with both natural and synthetic polymers in order to enhance the interaction between multiple polymers. This ultimately leads to improved performance of the drug delivery system in question. Moreover, the hydrophilicity of PVA makes it dominant over hydrophobic fibers as hydrophilicity enhances the drug release characteristics and drug loading efficiency [13]. The publication data for PVA-based drug delivery systems using the electrospinning technique and PVA-based drug delivery systems are shown in Figure 5a,b, respectively. The data for Figure 5a were extracted from Web of Science (WoS) based on the keywords “drug delivery”, “electrospinning”, and “PVA/Polyvinyl Alcohol” while for Figure 5b, the keywords “drug delivery” and “PVA/Polyvinyl Alcohol” were used. The data are presented until 2022, and 28 papers have already been published in 2023 in the field of electrospinning-based drug delivery systems. The WoS data suggest that PVA has been utilized for the development of drug delivery systems since 1987; however, electrospun PVA mats were first reported in 2005. Along with other properties, the electro-spinnability of PVA increases its potential in the development of DDSs which require the fibrous matrix as a drug carrier. The utilization of PVA in electrospinning-based drug delivery systems is discussed in the following sections.

### 3.1. Transdermal Drug Delivery Systems (TDDSs)

Transdermal drug delivery systems are designed to administer a drug formulation topically onto undamaged skin, allowing the drug to permeate through the various layers of the skin (including the epidermis and dermis) and reach the dermal layer without any accumulation, thereby facilitating systemic absorption. TDDSs are also utilized as a substitute for parenteral routes [49]. 

**Figure 6:**
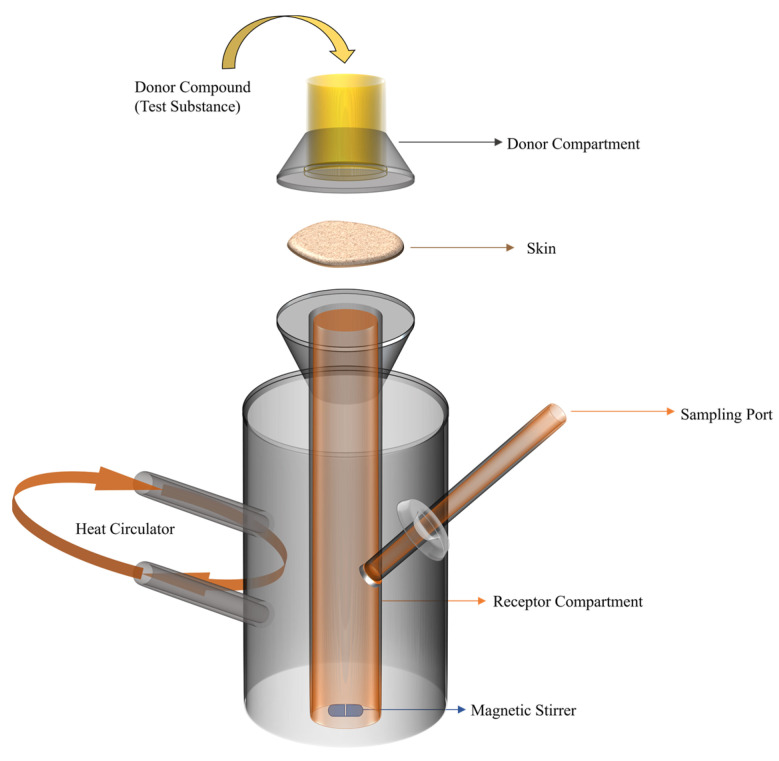
Schematic of Franz diffusion cell for transdermal DDS analysis.

A Franz diffusion cell is used to analyze the in vitro study of drug diffusion through the skin for transdermal drugs. Figure 6 shows the schematic of the Franz diffusion cell. The drug-loaded fiber mats (transdermal patch) are the donor compounds that release the drug through skin/skin-like membranes to the receptor compartment. The receptor compartment is filled with the drug release media/buffer solution which is examined for estimated drug release after a certain time.

PVA is a hydrophilic polymer with a semicrystalline structure, exhibiting excellent electro-spinnability. Additionally, it demonstrates favorable chemical and thermal stability. Given its biodegradability, non-toxicity, and other mentioned characteristics, polyvinyl alcohol (PVA) has been extensively researched both on its own and in combination with natural and synthetic polymers for the development of electrospinning-based TDDSs. 

When drug-loaded PVA fibrous mats are used in a transdermal DDS, there are a few factors associated with the drug release, including the MW of the drug, its concentration, and the surface area of the drug carrier [50]. Numerous studies have been conducted on drug-loaded polyvinyl alcohol (PVA) fibers, investigating the impact of different factors on their morphology, physical characteristics, and drug release properties in transdermal drug delivery systems (TDDSs). Significant research studies involving electrospun PVA-based TDDSs are discussed below. 

**Figure 7:**
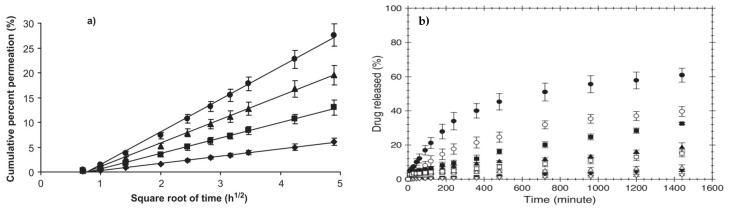
Effect of the molecular weight of a drug on (**a**) cumulative percent permeation (%), *MX* loading concentration at (♦) 2.5%, (■) 5%, (▲) 10%, and (●) 20 wt% of PVA (reprinted with permission from Taylor & Francis Online) and (**b**) the drug release (%) profile of (●) *sodium salicylate*, (▲) *diclofenac sodium*, (▼) *indomethacin*, and (■) *naproxen* drugs released from drug-loaded electrospun PVA mats (closed symbols) and as-cast PVA films (open symbols) by the transdermal diffusion through a pig skin method over a period of 0–1440 min (reprinted with permission from IOP Science) [51,52].

T. Ngawhirunpat et al. reported the effect of the amount of drug loaded in electrospun PVA fibers (uniaxial electrospinning) in a transdermal drug delivery system. *Meloxicam (MX)* was used as the model drug, which is a non-steroidal anti-inflammatory (NSAID) drug used for controlling pain and inflammation in rheumatic disease. In this research study, shed snakeskin was used as permeation media in a Franz cell to analyze the drug release. It was reported that the amount (wt%) of the loaded drug directly affects the release rate. The increased drug release rate with an increase in %*MX* in a polymer matrix is attributed to a decrease in the relative amount of polymer as a diffusion barrier. Therefore, the higher concentration gradient led to enhanced permeation of the drug (Figure 7a). This study suggests that *MX-*loaded PVA fibers have great potential in therapeutic transdermal DDSs [51].

P. Taepaiboon et al. reported four model drugs loaded in PVA fiber mats to analyze the different parameters that affect the morphology and drug release characteristics of the electrospun mats [52]. The NSAID drugs used for this purpose included *Sodium salicylate* (*SS*), *diclofenac sodium* (*DS*), *naproxen* (*NAP*)*,* and *indomethacin* (*IND*). Uniaxial electrospinning was used for the preparation of the fibrous mats at a 15 kV/15 cm electrostatic field strength. Swelling of the hydrogels is an important factor in controlled drug release and the degree of swelling in this case was calculated using the following mathematical expression:

(1) $$Degree\;of\;Swelling\;(\%)=\frac{M-M_{d}}{M_{d}}\times 100$$

where *M* and *M_d_* correspond to the weight of the sample after submersion into the buffer solution for 24 h and the weight of the dry sample after its submersion into the buffer solution for 24 h, respectively. 

The morphological analysis of the samples revealed high bead density for the fibers loaded with *SS* and *DS.* This can be attributed to the excessive enhancement in the conductivity of the respective solutions, which ultimately impacted the surface tension. However, for *NAP-* and *IND-*loaded solutions, bead-free fibers were formed. The viscosity in both cases increased; however, the effect of their addition in the spinning solution on conductivity and surface tension was negligible. The thermal stability was also studied, which revealed that *NAP* and *IND* incorporation enhanced the thermal stability of the PVA matrix as compared to those loaded with *SS* and *DS*. Figure 7b shows the cumulative drug release plots for all samples. The findings of the study provided confirmation that drugs possessing higher molecular weights exhibited a slower rate of drug release in comparison to those with lower molecular weights. It should be mentioned that the high water solubility of *SS* and swelling of the PVA hydrogel matrix are attributed to the burst release of the drug. The drug released through pig skin from electrospun PVA matrix was 60, 33, 19, and 5% for *SS* (160.1 g/mol), *NAP* (230.3 g/mol), *DS* (318.1 g/mol), and *IND* (357.8 g/mol), respectively. The immersion drug release method showed similar results with respect to the molecular weights of the loaded drugs in electrospun PVA mats.

Recently, a combination of electrospinning and cryogelation (freeze–thaw cycle) to create a dual PVA patch for applications in TDDSs was reported. Freeze-and-thaw cycles have proven to be beneficial for the enhancement of the mechanical properties of polymer fibers. The higher number of freeze and thaw cycles leads to improved crystallinity and stability of the dual PVA layers, which eventually improves the tensile strength [53].

In another research study by Sa’adon et al., electrospun (2 mL and 3 mL solution) PVA (10 wt% in water) fiber mats were prepared and a *DS* (0.0 (un-medicated), 1.0, 1.5, and 2.0%)-medicated PVA layer was added on the electrospun PVA mats [54]. The cryogelation of the dual-layered samples was carried out via three freeze–thaw (24 h freeze at −20 °C, 2 h thaw at room temperature) cycles. The medicated samples were analyzed for their in vitro drug release properties by Franz diffusion cell. The swelling capacity of the samples was estimated in terms of the equilibrium swelling ratio (ESR) by the given formula.

(2) $$ESR\;\left(\%\right)=\frac{W_{t}-W_{i}}{W_{i}}\times 100$$

where *W_i_* and *W_t_* represent the weight of the dual layer before and after swelling, respectively.

The cross-sectional analysis of the non-medicated PVA patches showed good polymer interaction between both layers due to the hydrophilicity of the PVA. However, it was also observed that the dual-layer thickness was higher for the PVA mat prepared using the 3 mL electrospinning solution. The swelling capacity of the dual-layer patches tends to decrease with an increase in *DS* loading content which results in narrow cryogel pores. Therefore, the increase in the loading content of *DS* decreased the water-absorbing capacity of the dual-layer patches.

**Figure 8:**
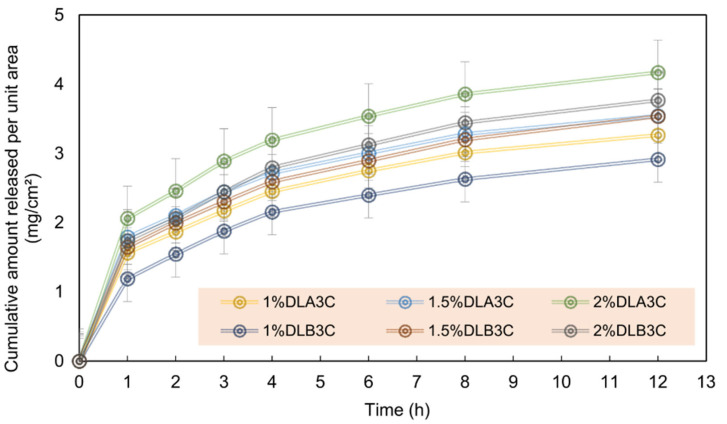
Cumulative amount of *DS* release per unit area (mg/cm^2^) for *DS*-medicated DL_A_3C and DL_B_3C [54].

Figure 8 shows the cumulative drug release (mg/cm^2^) for each medicated sample with the nanofiber side of the dual patch facing the cellulose nitrate membrane. The subscripts A and B in the nomenclatures (%DL_A_3C and %DL_B_3C) indicate the cryogels added on the electrospun PVA mats prepared using 2 mL and 3 mL electrospinning solutions, respectively. Considering the 1% *DS-*loaded dual-layers 1%DL_A_3C and 1%DL_B_3C, the drug releases for the first hour were 44.23% and 33.65%, respectively. After 12 h of diffusion, the *DS* release amounts for DL_A_3C (1, 1.5, and 2.0%) were 92.31%, 66.60%, and 58.94% while for DL_B_3C they were 82.40%, 66.47%, and 53.26%. These results indicated that the drug release was inversely related to the amount of the loaded *DS* drug and the thickness of the PVA fiber mat. Specifically, the dual-layer PVA patches loaded with 2% *DS* demonstrated the potential for extended drug release lasting up to 24 h.

There are a few other studies that suggest that the drug-loaded PVA fibers have shown promising results in the field of transdermal drug delivery systems. G. Teng et al. reported the prolonged drug release (72 h) of *Lappaconitine* trifluoroacetate (LAF)-loaded PVA fiber mats. *LAF* is a new derivative of Lappaconitine which is a potent analgesic drug. This study suggested that the *LAF*-loaded PVA mats can be a potential treatment for chronic and long-term pain [55]. 

Chitosan is a widely utilized polymer in conjunction with polyvinyl alcohol (PVA) for the advancement of drug delivery systems. Some significant studies involving chitosan/PVA are discussed here. Z. Cui et al. reported a drug-loaded chitosan (CS) and PVA (crosslinked) composite fiber matrix for transdermal drug delivery [56]. Uniaxial electrospinning was utilized for fiber fabrication. The crosslinking of the two polymers was performed using glutaraldehyde (GA). The effect of crosslinking on the morphology, chemical structure, thermal and mechanical behavior, hydrophobicity, and drug release, was studied. An amount of 10% of *ampicillin sodium* was used as a model drug to study the in vitro drug release. Moreover, the effect of drug concentration on the morphology and drug release properties was also studied. The drug release was analyzed by the Korsmeyer–Peppas model:

(3) $$\frac{M_{t}}{M_{\infty }}=Kt^{n}$$

where $\frac{M_{t}}{M_{\infty }}$ is the fraction of drug released at a time *t*, *K* is the rate constant, and *n* is the release exponent. The drug release mechanism is defined by Fickian (*n* ≤ 0.45) and non-Fickian diffusion (0.45 < *n* < 0.89). 

**Figure 9:**
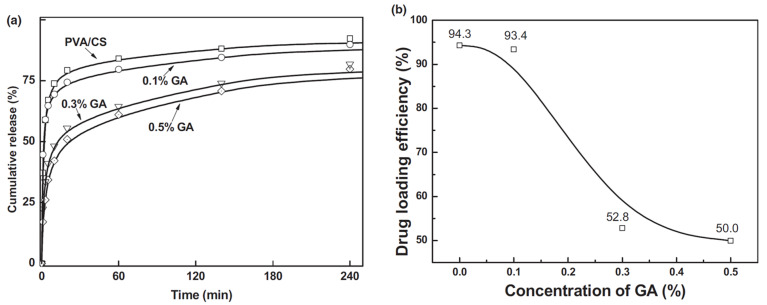
Effect of (**a**) %GA crosslinking of PVA/CS on cumulative drug release (%) as a function of time (min), (□) represents non-crosslinked PVA/CS fibers, (◦) represents 0.1% GA crosslinked PVA/CS fibers, (∇) represents 0.3% GA crosslinked PVA/CS fibers, and (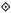) represents 0.5% GA crosslinked PVA/CS fibers and (**b**) concentration of GA (%), on drug loading efficiency (%) [56].

The morphological analysis confirmed the bead-free formation of fibers before and after crosslinking. The diameter of the fibers decreased due to an increase in CS content, as it increased the conductivity of the solution. The crosslinking of CS/PVA fibers enhanced the mechanical strength and thermal stability of the composite fibers. Surface wettability is one of the significant factors in determining the drug release rate of a DDS. The GA crosslinking of CS/PVA improved the hydrophobicity of the composite fibers because of -OH and -NH_2_ consumption. Enhanced hydrophobicity effectively mitigated the occurrence of rapid drug release, thereby offering advantages for the implementation of controlled drug delivery systems. These results were further verified by an in vitro drug release study. Figure 9a shows the drug release profiles of loaded *ampicillin sodium* CS/PVA (crosslinked and non-crosslinked). The burst effect of CS/PVA composite fibers was found to be 63%, but this was significantly reduced to 23% through the application of 0.5% GA crosslinking. The drug release was analyzed using the Korsmeyer–Peppas model, and the value of n was determined to be 0.34, indicating a Fickian diffusion mechanism. The drug loading efficiency (Figure 9b) shows decrement with an increase in %GA crosslinking, which is attributed to a reduction in the volume space of the interpolymer chain.

Another study evaluated the chitosan-*Thiamine Pyrophosphate*(CS-*TPP*)/Polyvinyl Alcohol (PVA) fiber mats for their potential in transdermal drug delivery systems [57]. This study suggested that increasing the amount of CS-*TPP* content increases the conductivity of the solution and leads to an increase in the bead density. CS-*TPP*/PVA fiber mats were suggested to be non-toxic and biocompatible, with good potential in transdermal drug delivery applications. 

R. Najafi-Taher et al. reported the transdermal drug release properties of *L-ascorbic acid (ASC)* drug-loaded CS/PVA core–shell fibers (coaxial electrospinning) and uniaxial CS-PVA/*ASC* fibers [58]. Both fibrous mats were crosslinked using the GA vapor method. For core–shell fibers, PVA and chitosan were used as shell material while *ASC* constituted the core. The crosslinked core–shell fiber mats showed a decreased drug release rate with an increase in CS content in the shell and a reduction in *ASC* content in the core. Such characteristics make the crosslinked core–shell PVA-CS/*ASC* fibers a good candidate for transdermal drug delivery. 

G. El Fawal reported the use of drug-loaded PVA/Hydroxyethylcellulose electrospun scaffolds for a transdermal drug delivery system [59]. The *fluorescein isothiocyanate (FITC)* loaded on ethosome (*FITC*@Eth) was the model drug for this study. The in vitro drug release analysis for these scaffolds suggested that the increase in ethosome from 26.5% to 43.5% can improve the cumulative drug release rate of the *FITC*. 

In the field of biomedical applications, the combination of collagen and nanocellulose, particularly cellulose nanofibril (CNF)-based composites, presents numerous opportunities for the development of biomaterials [60,61]. Recent research has investigated the potential of utilizing nanocollagen (NCG) as a graft material on the surface of CNF for biopharmaceutical purposes [60]. An interesting study suggested the use of a NCG-CNF drug prepared from NCG extracted from fish scales and CNF isolated from the jute fibers [62]. Jute fibers possess a high degree of efficiency in the extraction of cellulosic fibers, which in turn exhibit a wide range of potential applications. NCG-CNF-reinforced PVA/methylcellulose (MC)/PEG GA-crosslinked fibers were studied for their potential in TDDSs. *Ketorolac tromethamine* (*KT*) was utilized as the model drug. The effect of NCG-CNF wt% on the drug release properties of NCG-CNF-reinforced fibers loaded with *KT* was studied. PVA/MC/PEG fibers with 1 wt% of NCG-CNF as a reinforcement agent showed the best results for in vitro drug release. The Fickian diffusion mechanism was observed for this formulation, which indicates the good interfacial interaction between the polymer matrix and the drug. 

Synthetic polymers such as PEG, PLGA, and PLA have widely been explored for polymeric drug delivery systems in transdermal drug delivery, cancer therapy, tissue engineering, and wound healing [63,64,65,66]. Electrospun PVA blended with synthetic polymers has also been extensively studied for TDDSs.

The use of electro-sensitive hydrogels as matrices for transdermal drug delivery systems (TDSs) has been extensively studied due to their ability to swell under the influence of an electric field [67]. J. Yun et al. explored the multiwalled carbon nanotubes (MWCNTs)/PVA/poly acrylic acid (PAA) hydrogel nanofibers for electro-responsive drug delivery [68]. The effect of the surface modification of MWCNTs was performed by oxyfluorination to improve the drug release characteristics of drug-loaded composites. *Ketoprofen* was used as a model drug. It was observed that the conductivity of the composites increased with MWCNTs. Due to variations in the ionization of the functional groups in the polymer matrix, swelling and drug release were found to vary dependently with the applied voltage (≤10 V), which confirmed the electro-responsive behaviors of prepared composites.

**Figure 10:**
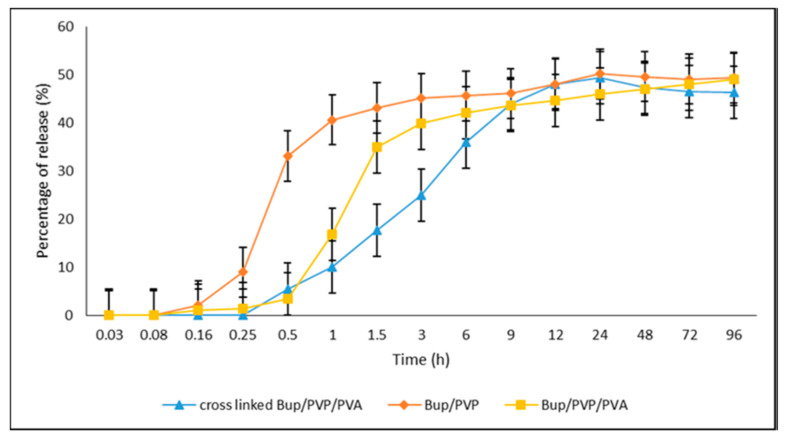
The drug release profile for buprenorphine with different polymer fiber formulations and their crosslinking [70].

Polyvinylpyrrolidone (PVP) is a synthetic polymer that lacks a crystalline structure and exhibits a strong attraction to water, as well as excellent adhesion properties. It is widely recognized as a crucial material in the field of biomedical and pharmaceutical applications due to its minimal chemical toxicity and compatibility with biological systems [69]. PVP/ PVA uniaxial fibers loaded with *buprenorphine* (*Bup*) were studied for their drug release characteristics. The effect of GA crosslinking on PVP/PVA/*Bup* composites on cumulative drug release (%) was also analyzed by using high-performance liquid chromatography (HPLC). The GA crosslinking prolonged the drug release as compared to the non-crosslinked composites with and without PVA. The crosslinked PVP/PVA/*Bup* composites have proven to be potential candidates for transdermal drug delivery patches for pain relief and controlled drug release [70]. Figure 10 shows the cumulative drug release (%) as a function of time for all the composites studied.

In conclusion, electrospun PVA fibers combined with a variety of natural and synthetic polymers have been effectively utilized for the development of transdermal drug delivery systems. The release of the drug is significantly influenced by the molecular weight and weight percentage of the drug incorporated into the fibers. The hydrophilic properties of PVA play a crucial role in facilitating drug release by enhancing the swelling behavior of the drug-loaded fibers. Additionally, the freeze–thaw cycle technique has been employed to improve the interfacial interaction between the polymers, resulting in enhanced drug-release properties of the respective formulations. However, it is important to note that there is a limited number of studies investigating coaxial and triaxle drug-loaded PVA fiber meshes. Furthermore, multi-layered fibrous mats necessitate further exploration in this particular research area. 

### 3.2. Electrospun PVA for Wound Dressing/Tissue Engineering

**Figure 11:**
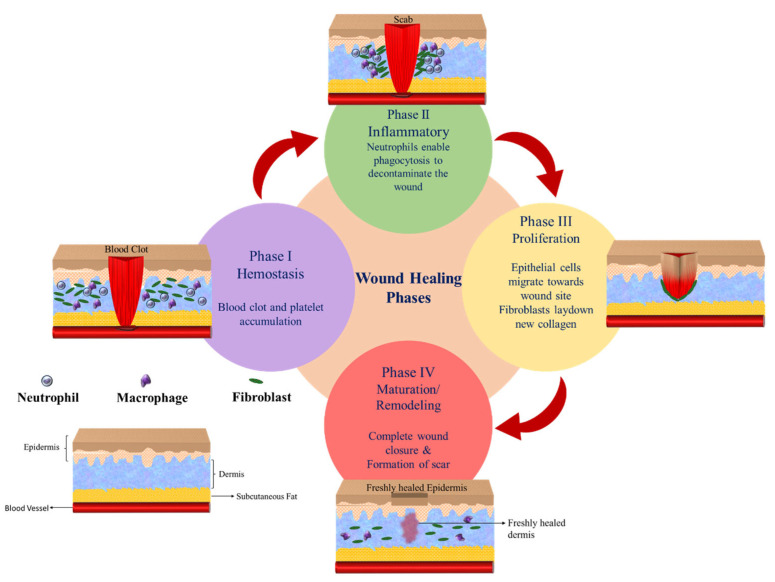
Skin layers and different phases of wound healing.

The most common use of PVA fibers in drug delivery applications is for wound dressing and tissue regeneration. Electrospun fibrous mats mimic the extracellular matrix (ECM) and provide the ideal microenvironment for wound healing [71,72,73,74,75,76]. High surface area, porosity, and interconnected pores of 3D fibrous mats enhance the cell attachment and gas exchange, and supply water and nutrients to the wound site [42,74,77,78,79]. Moreover, fibrous mats aid the fluid absorption and balance the wound moisture [71,72,79]. In the realm of medical terminology, wounds are defined as injuries that affect the epidermal and/or dermal layers of the skin. These wounds can be further classified into two distinct categories based on their healing duration: acute wounds and chronic wounds. The healing time (4–12 weeks) for acute wounds depends on their intensity, depth, and size; however, if not treated properly, they can become chronic. Chronic wounds encompass various conditions such as burns, skin ulcers, and diabetic wounds. The healing process of a wound is typically characterized by four distinct phases: hemostasis, inflammation, proliferation, and maturation/remodeling phase, as illustrated in Figure 11. However, for chronic wounds, the healing process is complex, especially in the inflammation and proliferation phases. The hemostasis phase starts immediately with the wound stopping the bleeding by promoting blood coagulation, along with the inflammation phase. The debris is removed in the inflammation phase to avoid microbial invasion and the epithelial cells occupy the site of injury to replace the dead cells. The granulation tissues are developed in the proliferation phase. Diabetic wounds, however, remain in the inflammatory phase, impeding the mature granulation tissues, resulting in a reduction in the injury tensile strength, which leads to ischemia. In the maturation or remodeling phase, the wound is covered by fibroblasts, and due to tissue remodeling a new epidermal layer is developed [80].

When designing a polymeric wound dressing, several crucial factors are taken into consideration. These factors include the ability to absorb water, the level of porosity and swelling, the permeability to gases, the presence of effective antimicrobial properties, the possession of strong mechanical strength, and the capacity to transport bioactive agents [80,81,82,83].

**Figure 12:**
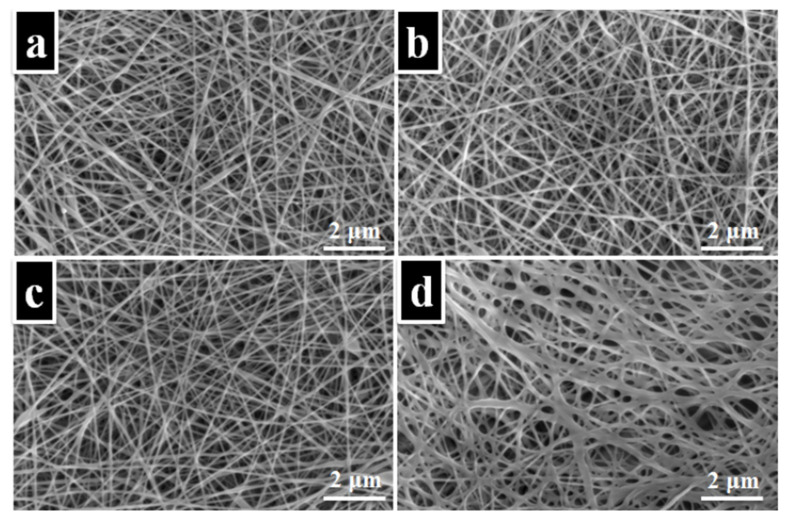
SEM micrographs for the electrospun membranes of (**a**) PVA, (**b**) PVA–*Thymus vulgaris*, (**c**) PVA–*Salvia officinalis folium*, and (**d**) PVA–*Hyperici herba* [85].

Given the desirable characteristics of PVA that are in line with the specifications of wound dressings, it has been extensively employed in the fabrication of electrospun wound dressings. PVA with different drug formulations has been reported by various research groups for its wound-healing potential. A. García-Hernández et al. reported PVA-based biomembranes with hydrolyzed collagen (HC) and *ethanolic extract* for their wound dressing characteristics [84]. Different concentrations of *ethanolic extract* of *Hypericum perforatum* (*EEHP*) in PVA/HC were evaluated. Uniaxial electrospinning was performed in a controlled environment. The higher concentrations of (8% 6%, and 32%) *EEHP* in 6%PVA/5%HC biomembranes exhibited excellent anti-inflammatory characteristics. The samples with no HC and 16% *EEHP* concentration in PVA/*EEHP* showed better antimicrobial activity against *Staphylococcus aureus* (*S. aureus*) and *Staphylococcus epidermidis* (*S. epidermidis*) microorganisms. The formulation PVA/5%HC/16%*EEHP* inhibited *S. aureus* only. Another innovative drug delivery system comprised of PVA fibers loaded with phytotherapeutic agents (such as *Thymus vulgaris*, *Salvia officinalis folium*, and *Hyperici herba* extracts) was reported by D. Serbezeanu et al. [85]. The antibacterial activity of the formulations against four bacterial strains, i.e., *S. aureus* ATCC 25923, *Methicillin-resistant Staphylococcus aureus* (*MRSA*) ATCC 33591 (Gram-positive), and *Escherichia coli* (*E. coli*) ATCC 25922 (Gram-negative) was investigated. All formulations showed promising antibacterial activity against the mentioned bacterial strains. Figure 12 below shows the SEM micrographs for PVA with each phytotherapeutic agent. Distinct fine fiber morphology was observed for all formulations, except for PVA/Hyperici herba extracts which were believed to collapse during the evaporation following the electrospinning. The promising findings suggested the significant potential of phytotherapeutic agents in PVA fibers for wound dressing applications. 

As mentioned earlier, emulsion electrospinning is utilized for core–shell fiber mats and holds great significance in controlled drug release applications. *Chelidonium majus L.* (*C. majus*)*-*incorporated emulsion electrospun fiber meshes have been reported for wound dressing applications. *C-majus-*encapsulated PCL/PVA-Pectin(PEC) was studied for its antimicrobial activity [86]. Fine morphology of the meshes was observed before and after the incorporation of the model drug. Highly porous *C-majus*-incorporated fiber meshes with 400% swelling ability were formed possessing a mechanical strength comparable to that of native human skin. The drug release analysis was performed for 30 days and showed 65.70 ± 4.13% *C. majus* release during the test period. The antimicrobial analysis confirmed the effective inhibition behavior of the prepared meshes against *S. aureus* (99.98 ± 4.43%, 3.82 log reduction) and *P. aeruginosa* (95.26 ± 5.52%, 1.32 log reduction) without compromising the cell viability. The analyses confirmed that the prepared formulation can potentially be utilized as antimicrobial wound dressings with accelerated wound healing. 

Wound dressings are developed by incorporating bioactive agents into a combination of natural and synthetic polymers, tailored to the specific type of wound. As mentioned earlier, CS is one of the most used natural polymers to develop DDS, however, it has its limitations in electro-spinnability due to its polycationic nature in solutions for electrospun drug delivery systems. Therefore, CS is combined with polymers that are electro-spinnable, biocompatible, biodegradable, non-toxic, and possess high mechanical strength. As mentioned earlier, PVA holds dominance in synthetic polymers due to its high mechanical strength, electro-spinnability, and the desired characteristics for a DDS. Moreover, PVA possesses good adhesion and high barrier properties for oxygen and odor, which makes it a prominent candidate for wound healing applications. The combined qualities of both PVA and CS make them an outstanding combination for sustained drug release and improving the wound healing rate. There are several factors associated with the CS/PVA electrospinning parameters that require optimization to prepare continuous, porous, and bead-free nanofibers. Such morphological properties play a significant role in drug-loaded CS/PVA composite nanofibers. G. Mata et al. reported a systematic study on the effect of composition on the morphological properties of CS/PVA electrospun nanofibers [87]. The electrospinning parameters after optimization were set at 20 kV (applied voltage), 0.5 mL/h (flow rate), and 10 cm (needle-to-collector distance). It was suggested that an electrospinning solution comprising 6% PVA and 1% CS led to a fine and bead-free morphology of the CS/PVA fibers. More than 2% CS increases the viscosity of the solution due to strong hydrogen bonding between the NH_2_ and OH groups of CS chains. The incorporation of polyvinyl alcohol (PVA) into chitosan (CS) enhances the intermolecular interactions between the two polymer chains, thereby reducing the interactions among CS chains. This phenomenon ultimately facilitates the process of solution spinning by reducing the extent of CS chain interactions. Increasing the CS content (%) in the solution hinders the porosity of the fibers. 

**Figure 13:**
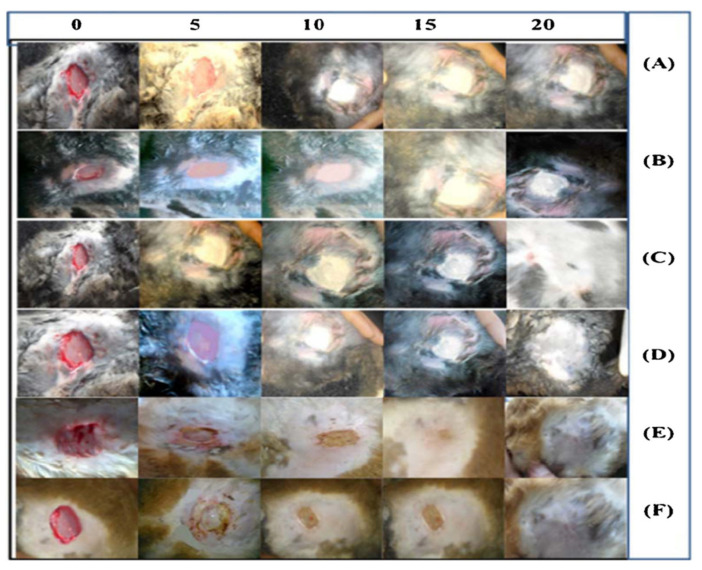
In vivo wound healing by different formulations: (**A**) control group, (**B**) PVA nanofibers, (**C**) PVA-NaAlg nanofibers, (**D**) drug-loaded PVA nanofibers, (**E**) drug-loaded PVA-NaAlg nanofibers, and (**F**) a marketed drug formulation (reprinted with permission from Elsevier) [94].

Several recent studies with significant potential in the field of wound dressing applications are examined in this discussion. There are several studies where CS and PVA have been used to prepare drug-loaded electrospun fiber mats for wound dressing applications. A. Stoica Oprea et al. explored CS, PVA, and *usnic acid* (*UA*) nanofibrous mesh for wound healing potential [88]. *UA* has been extensively explored for its healing properties for burns and wounds [89,90]. Uniaxial electrospinning was used to prepare a 5%PVA/2%CS/1%*UA* nanofibrous mesh. The SEM analysis showed the high porosity of the prepared samples. The mesh mimicked the ECM, which is ideal for wound healing applications. The cell viability was increased to 30% after 48 to 72 h, which demonstrated its suitability as a biodevice to sustain cell proliferation and growth with no indication of cytotoxicity. The reported formulation emerged as an ideal candidate for wound healing applications. Various materials have been explored to enhance the biocompatibility and performance of DDSs. Graphene oxide is one such material as it possesses unique physiochemical properties and biocompatibility. However, when used with CS, it requires surface modification to enhance its compatibility [91]. S. Yang et al. reported GO/CS/PVA nanofibrous membranes loaded with *Ciprofloxacin* (*Cip*) and *Ciprofloxacin hydrochloride* (*CipHcl*) to study their wound healing efficiency [92]. The drug release analysis of GO/CS/PVA/*Cip* and GO/CS/PVA/*CipHcl* showed that GO promoted the drug release ratios and assisted in avoiding the initial burst release. The presence of GO nanosheets increased the distance between the nanofibers, which led to an increase in the drug release ratio, and after 168 h GO/CS/PVA/*Cip* and GO/CS/PVA/*CipHcl* showed 96.5% and 62.1% drug release ratios, respectively. In terms of antibacterial activity, the prepared fibrous mats were effective against *E. coli* and *B. subtilis*; however, they did not show promising results against *S. aureus*. Furthermore, H. Iqbal et al. reported the antibacterial activity of *Cefadroxil* (*CFX*)-loaded CS/PVA fiber mats against *Staphylococcus aureus* (*S. aureus*) [93]. The SEM analysis showed the bead-free formation of the drug-loaded CS/PVA fibers. Prolonged (~13 h) and sustained antibiotic drug release was reported with *CFX*-loaded CS/PVA fiber mats. A non-Fickian diffusion mechanism (*n*~0.513) was observed for the drug release. K. Kataria et al. reported on in vivo wound healing using the antibiotic *ciprofloxacin* (*CPFX*)-loaded PVA/NaAlg (sodium alginate) [94]. The effect of different formulations (PVA fibers, PVA/NaAlg, *CPFX*-loaded PVA fibers, and *CPFX*-loaded PVA/NaAlg) on the in vivo wound healing was studied. The *CPFX* was found to be compatible with the PVA/NaAlg and exhibited controlled drug release properties following the non-Fickian diffusion mechanism (*n*~0.7714). The wound healing rate for *CPFX*-loaded PVA/NaAlg was observed to be the highest as compared to the rest of the formulations. Figure 13 shows the in vivo wound healing for all the formulations reported in this research study.

In another study, co-electrospun CS/PVA and silk fibers (SFs) were fabricated for skin regeneration characteristics [95]. The SFs were seeded with differentiated keratinocytes. The hybrid fiber structure showed superior mechanical strength and biocompatibility with desirable swelling and hydrophilic characteristics. Moreover, cell adhesion and proliferation were found to be excellent for the prepared hybrid fiber samples. In vivo, the wound healing profile showed that the presence of differentiated keratinocytes stimulates the wound healing and skin regeneration process. Another innovative drug delivery system was developed by modifying the CS for CS/PVA fibrous mat encapsulated with indomethacin as a model drug by using the uniaxial electrospinning technique [96]. For the surface modification of CS, *β-cyclodextrin*-grafted (*β-Cd*-g) CS was obtained to improve its spinnability. *β-Cd*-g-CS/PVA fibers showed high mechanical strength and a bead-free fiber morphology. Fast swelling was observed for the prepared samples, which is advantageous for controlled drug release. The swelling behavior of the samples observed in this study aligns with the drug release process, suggesting that the mechanism of drug release is controlled by swelling. As compared to the CS/PVA fiber matrix, the drug loading efficiency and sustained drug release characteristics for *β-Cd*-g-CS/PVA were found to be superior. Initial drug release was found to be faster for *β-Cd*-g-CS/PVA fibers, which is associated with four factors, i.e., higher drug solubility in polymer, increase in drug diffusivity, higher drug accumulation in the polymer matrix, and enhanced solubility of *β-Cd*-g-CS/PVA. 

**Figure 14:**
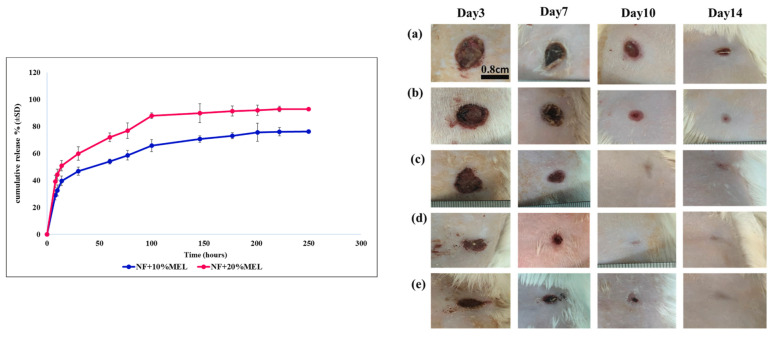
(Left) cumulative drug release of *melatonin* (with two concentrations of NF + 10% *MEL* and NF + 20% *MEL*) from three-layered nanofibers; (right) gross image of the nanofiber wound dressing on days 0, 3, 7, 10, and 14 post-wounding: (**a**) non-treatment (free gauze), (**b**) NF, (**c**) NF + 10% *MEL,* (**d**) NF + 20% *MEL*, (**e**) *Comfeel* Plus. Scale bar: 0.8 cm (reprinted with permission from Elsevier) [97].

In a notable research endeavor, an investigation was conducted to analyze the effects of drug-loaded PVA that was sandwiched between PCL and CS polymers, both in vivo and in vitro [97]. Eight different formulations for PCL/CS were electrospun to analyze the accumulative effect on drug release and wound healing properties of the samples. The middle layer with PVA/*melatonin* (*MEL*) was prepared with two different formulations, i.e., 10 wt% PVA and, 10 and 20 wt% *MEL* drug. The porosity of the nanofibers is a critical factor in the release of drugs and the enhancement of wound healing. Consequently, the outer layer consisting of 10 wt% CS/6 wt% PCL aligns with the necessary specifications, exhibiting an average diameter of 103 nm and a porosity of 55%. The in vitro and in vivo study was carried out with 10 wt% CS/6 wt% PCL as outer layers of PVA with 10 and 20% *MEL*. Hydrophilicity plays a major role in designing a wound dressing as it enhances the wound healing rate by providing optimum moisture to the wound site. Nanofibers (NFs) were the drug release medium and with the degradation of PVA with time, the porous outer PCL/CS layers aided the drug release. Along with the hydrophilicity advantage, PVA also played a role in the homogeneous distribution of drugs in the medium layer, which assisted in sustained drug release. NF + 10% *MEL* showed burst release (44%) for the initial 14 h and NF + 20% *MEL* exhibited 51% burst release. Both formulations showed sustained release for 250 h. The in vivo wound healing showed promising results for NF + 20% *MEL* as it accelerated the wound healing process. It exhibited the complete regeneration of the epithelial layer and the remodeling of the wound. Figure 14 shows the cumulative drug release of the prepared formulation and in vivo wound healing, respectively.

The coaxial electrospinning technique has also been used for the preparation of PVA-based potential wound-healing fiber mats. PVA–PEG–SiO_2_@PVA–GO core–shell fibers were prepared by Y. Kan et al. to evaluate their potential for wound dressings [98]. The PVA-PEG-SiO_2_ as the core and PVA-GO as the shell of the core–shell fibers were evaluated for their structural, mechanical, and swelling characteristics. The 10% GO in the shell enhanced the swelling and mechanical properties of the fibers after crosslinking while the incorporation of silica and PEG in the core enhanced the fiber morphology. The crosslinking increased the fiber diameter for all the formulations. The prepared formulation holds potential for wound dressings while overcoming the challenges associated with drug loading volume and film thickness. 

Considering the promising characteristics of core–shell nanofibers, M. Abdul Hameed et al. reported the potential of drug-loaded core–shell fiber crosslinked thermally with different biopolymers including CS, carboxymethyl cellulose (CMC), carboxymethyl starch (CMS), and hydroxypropyl cellulose (HPC) [99]. *Cephalexin* was used as a model drug and the PVA-to-biopolymer ratio was kept at 90:10. The emulsion electrospinning technique was used to prepare the core–shell fiber meshes. The fiber diameter was observed in the range of 270–526 nm for all the samples. In vitro drug release analysis revealed the release rate after 2 h as *PCH* (7%) < *PCMS* (15%) < *PHPC* (17.4%) < *PCMC* (19.5%). However, the drug release rate for PVA alone was 40% for the initial 2 h. Following the Fickian diffusion mechanism, a drug release trend was observed after 8 h with 10.65% for *PCH*, 23% for *PCMS*, 25% for *PHPC*, and 30% for *PCMC.*

**Figure 15:**
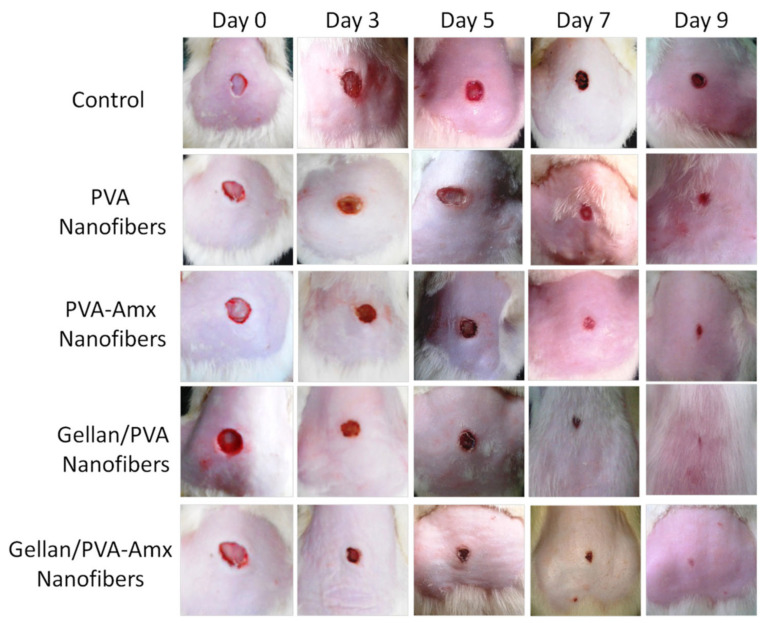
Wound healing evaluation of full-thickness excision wounds in rats. Macroscopic appearance of wound closure at 0, 3, 5, 7, and 9 days after treatment with different fabricated formulations (reprinted with permission from Elsevier) [105].

Gellan is another natural polymer that has been extensively utilized for the development of drug delivery systems [100,101,102]. However, similar to CS, gellan has electro-spinnability limitations mainly because of its limited solubility and highly viscous solution [103]. Furthermore, for solution prepared with minimal gellan concentration (1%) results in deformed droplets instead of fiber jet formation. This is due to the anionic nature of gellan, low or high low-shear viscosity, and strong shear thinning behavior at low shear rates [104]. Therefore, it is combined with polymers with high spinnability such as PVA. P. Vashisth et al. reported on the use of *amoxicillin* (*AMX*)-functionalized gellan/PVA uniaxial fiber mats for their wound healing potential [105]. The in vivo analysis of the transdermal drug release for wound healing on a rat model showed promising results. The wound closure rate was found to be 86% for *AMX*-functionalized gellan/PVA fibers. Figure 15 shows the in vivo wound healing of *AMX*-functionalized gellan/PVA fibers as compared to the untreated wound and the rest of the fiber mats in this study.

*Curcumin* (*CUR*) derived from *Curcuma longa* L., is a traditional wound healing pharmacological compound that possesses various therapeutic characteristics such as anti-tumor, anti-inflammatory, anti-bacterial, antidiabetic, and antioxidant capacities [106,107,108]. S. Rathinval et al. reported the amine-functionalized SBA-15-incorporated PVA/CUR nanofibers for their drug release properties to improve wound healing [109]. It was suggested that due to its biocompatibility and porosity, amine-functionalized SBA-15 not only improves the *CUR* solubility but also assists in its sustained drug release. Moreover, it enhanced the cell attachment characteristics, biocompatibility, and antibacterial activity (against *E. coli* and *S. aureus*) of the mats. In vitro study showed 73% drug release in 80 h and in vivo study showed complete wound healing in 18 days. The histopathology of the samples was also studied, and it confirmed the formation of re-epithelization tissue, granulation tissue, and dense collagen deposition.

Using herbal extracts for wound healing has been studied for decades for their excellent antimicrobial activity. M. Salami et al. reported an innovative drug delivery system for wound healing. *Bitter gourd extract* (*Ex*) was used for its biological activities [110]. Different formulations of PCL/PVA/Collagen/(1%, 5%, 10%) *Ex* co-electrospun fiber mats were studied for their in vitro anti-bacterial and in vivo wound healing activities. The in vitro study showed characteristics such as swelling kinetics, bacterial penetration barrier, porosity, mechanical strength, and water vapor permeability. It indicated that these formulations provide a suitable environment for wound healing. Moreover, in vivo wound healing analysis showed that the incorporation of the bitter gourd extract promoted wound healing. The incorporation of 5% *Ex* and 10% *Ex* improved the formation of the epidermal layer. In another study, plant extracts *Galinsoga parviflora Cav* (*GP*) and *Cissus quadrangularis* (*CQ*) were loaded in PVA to study their antimicrobial activity against *S. aureus* and *E. coli* [111]. Different concentrations of *CQ* and *GP* (100:0, 70:30, 50:50, 30:70, and 0:100) were loaded in PVA to analyze their effect on the morphology, drug release, and antimicrobial activity of the fibrous meshes. An increase in *GP* concentration led to a decrease in the fiber diameter. The in vitro drug release analysis showed the following trend: 

PVA/*CQ:GP*(100:0) ˃ PVA/*CQ:GP*(70:30) ˃ PVA/*CQ:GP*(50:50) ˃ PVA/CQ:GP(30:70) > PVA/*CQ:GP*(0:100). 

The formulation PVA/*CQ:GP* (30:70) showed superior antimicrobial activity against *S. aureus* and *E. coli* while PVA + *CQ:GP* (70:30) exhibited promising inhibition of *E. coli*. Cell viability analysis showed that an increase in *GP* increases cell viability.

Polymers are blended with metal and metal oxide nanoparticles such as gold (Au), silver (Ag), platinum (Pt), and ZnO to improve their antibacterial effect [112,113]. 

A novel *Punica granatum* L. *extract* (*PE*)-loaded drug delivery system for wound healing was explored by M. Hussein et al. *PE* was combined with CS-Au (Gold) nanoparticles and was then loaded in PVA fibers [114]. *PE*-CS-Au/PVA composites were studied for their antimicrobial activity. The GA crosslinking of the PVA fibers was also performed which improved the mechanical strength, maintained the porosity, and enhanced the drug release characteristics of the composites. A Fickian diffusion mechanism was observed for the *PE* drug release trend. The studied formulation showed long-term stability, excellent cyto-biocompatibility, and strong cell adhesion and proliferation. 

As mentioned earlier, diabetic wounds are challenging to heal, and thus various studies have been reported to overcome these challenges. 

S. Yadav et al. reported the CS/PVA blended fibers loaded with curcumin (*CUR*) and zinc oxide (ZnO) to explore their potential in healing diabetic foot ulcers (DFU) [115]. In vitro drug release and in vivo wound healing activity of the fiber mats were analyzed. The *CUR*-ZnO-CS/PVA fiber mates were crosslinked. The fiber mats showed controlled drug release characteristics for 72 h. The non-toxicity of the prepared fiber mats was also confirmed by a cytotoxicity analysis. *CUR* and ZnO release against *S. aureus* and *P. aeruginosa* showed promising antimicrobial activity. The in vivo wound healing analysis showed sustained drug delivery to the wound site, which improved the wound contraction ability of the fiber mats for 14 days.

pH-responsive *CUR* release from PVA/GO-Ag-*CUR* nanofiber mats has been studied by E. Rahmani et al. [116]. Fine porous morphology of the prepared fibers was observed. The nanofibrous structure showed promising anti-bacterial activity against *E. coli* and *S. aureus* bacteria. Moreover, it assisted in the migration and proliferation of fibroblasts. The drug release of the prepared samples at pH 5.4 and 7.4 was studied for the prepared samples. Cumulative drug release was observed to be 95% for pH 5.4 and 64% for pH 7.4 within 96 h. Such drug release trends verified the pH-responsive characteristic of the PVA/GO-Ag-*CUR* nanofiber mats.

Sandoval-Herrera et al. explored the *chlorogenic* (*CGA*)-loaded PVA/Poly(γ-Glumatic Acid) (γ-PGA) blended electrospun fiber mats for diabetic foot treatment [117]. PVA/γ-PGA (5 and 10%) blended polymers were crosslinked using the GA vapor technique to enhance their drug release characteristics. The uniaxial electrospinning technique was used for the preparation of drug-loaded fiber mats. The higher blending percentage of γ-PGA led to a smaller fiber diameter and improved the drug release characteristics. The drug release for PVA/γ-PGA(10%) was observed to be 66% for the first hour and 82% after 72 h. Due to strong hydrogen bonding in the PVA and partial repulsion of γ-PGA to negatively charged carboxylic acids in the *CGA* improved the drug release properties. 

Further studies are presented in Table 2 showing the innovative drug delivery systems for wound healing using electrospinning-based PVA.

In summary, there has been significant research conducted on the use of electrospun drug-loaded PVA fibers for wound dressing purposes. Both uniaxial and coaxial electrospun fibers have been utilized, with the latter demonstrating a higher capacity for drug loading. The combination of PVA with natural and synthetic polymers has proven effective in promoting wound healing and combating antimicrobial resistance. Fibrous mats containing *CUR* have shown promising results in facilitating wound healing. Additionally, the incorporation of herbal extracts with PVA has also yielded positive outcomes. Despite the extensive investigation into the potential of PVA for wound healing applications, there is a dearth of literature on layered fibrous mats and triaxial fiber structures, which may possess exceptional antimicrobial resistance and wound healing capabilities.

### 3.3. Electrospun PVA for Tissue Regeneration

The field of organ and tissue replacement and regeneration has garnered considerable attention. In the realm of traditional medical treatment, the primary obstacles lie in the identification of a suitable donor and the potential rejection of the transplanted tissue or organ. Consequently, numerous investigations have been conducted to explore the potential of electrospinning in the creation of functional tissues capable of regenerating and enhancing damaged tissue [126,127,128]. Tissue engineering deals with medicinal techniques to repair and restore the structural functions of damaged tissues using scaffolds, cells, decellularized matrices, etc. Scaffolds are three-dimensional (3D) structures that mimic the ECM and can regulate cellular responses [45]. When developing a scaffold for tissue regeneration, several factors are taken into account. These factors encompass the biocompatibility, bioactivity, mimicry of extracellular matrix (ECM), bioabsorption, as well as sufficient mechanical and thermal strength [129,130,131]. An ideal scaffold will provide an adequate healing environment for damaged tissue by providing optimum moisture, a surface for cell adhesion, and interaction with the biomolecules of interest [132]. Natural and synthetic polymers are used to prepare ideal scaffolds for tissue regeneration [133]. PVA with various natural and synthetic polymers has vastly been used for bone and skin regeneration because of its superior physical properties and biocompatibility. 

**Figure 16:**
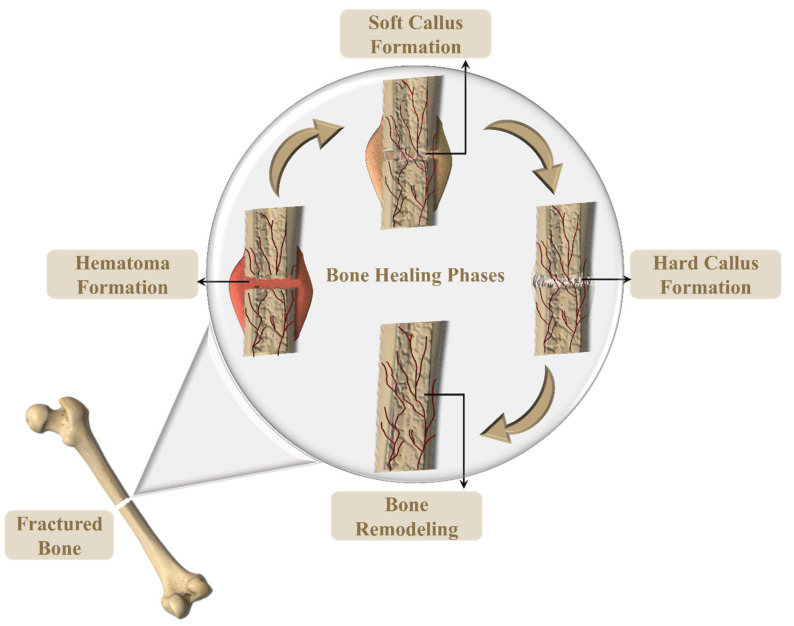
Bone healing phases.

As shown in Figure 16, bone regeneration is comprised of four phases including the hematoma (inflammation) phase, soft callus formation, hard callus formation, and bone remodeling [134]. Hematoma formation occurs when the blood clots the fracture site, followed by the formation of soft granulation tissue formation. The capillaries grow in the hematoma while the phagocytic cells play their part in removing the debris. Like the wound healing process, fibroblasts play a key role in fracture repair by invading the fracture site and producing the collagen fibers that eventually connect to the broken bone. Osteoblast cells aid in forming the spongy bone structure which is known as the soft/fibrocartilaginous callus. Hard callus replaces the soft callus which is detectable by X-ray after a few weeks of injury. The final phase as mentioned above is bone remodeling which takes months to complete [134].

There are various significant in vitro and in vivo studies that explored the electrospun scaffolds for bone regeneration. R. Boda et al. reported the *β-Tricalcium Phosphate-Modified Aerogel* (*BTCP-AE*) containing PVA/CS electrospun fiber mesh for their bone regeneration potential [135]. The focus of this study was to analyze facial bone regeneration and an in vivo study was carried out on calvarial defects in rats. The SEM analysis of *BTCP*-AE-PVA/CS showed the effect of crosslinking on the morphology of the fiber meshes. It was observed that the fiber diameter was in the range of 50–450 nm after crosslinking with an average diameter of 147 ± 50 nm, however prior to crosslinking the average diameter was observed to be 107 ± 22 nm. The morphology of the prepared samples exhibited an ideal surface for the dental pulp stem cell (DPSC) attachment. Moreover, the fiber meshes retained their hydrophilicity and were found not to have a cytotoxic effect on DPSCs which suggested their utilization in living cells. In vivo study showed the promising osteo-inductive feature of the meshes. An 80% ossification of the bone defect was observed of up to 6 months duration. Polymeric drug delivery systems have also been explored for their potential in Periodontitis treatment. It is an inflammatory disease that damages the periodontal tissues. CS/PVA core–shell nanofibers were evaluated by D. dos Santos et al. for their potential in treating periodontitis. The coaxial electrospinning technique was used to prepare CS/PVA core–shell fibers [136]. *Tetracycline hydrochloride* (*TH*) was combined with PVA as the core of the fibers and CS was attributed to the formation of the fiber shell. CS was processed through an average degree of deacetylation (D͞D) and electrospun fibers were crosslinked using genipin to analyze its effect on the physiochemical and biological properties of the core–shell fibers. Bead-free fine fiber morphology for all the samples was observed. The genipin crosslinking of the fibers led to a decrease in fiber diameter, hydrophilicity, degradation, and swelling ratio, along with enhanced mechanical strength. The in vitro drug release analysis revealed the sustained drug release over a period of 14 days for crosslinked core–shell fibers. Moreover, the crosslinking of the core–shell fibers promoted their cytocompatibility. The anti-bacterial activity of *TH* against periodontal pathogen was promising and all these characteristics aligned with the requirements for effective periodontitis treatment. 

The application of inorganic ceramics, such as silica, in bone tissue engineering has been investigated due to their exceptional thermal resistance, high rigidity, and wear resistance, as well as their resistance to oxidative and chemical degradation [137]. Most recently, silica has been utilized for its potential in bone tissue restoration/repair. According to research findings, this material is characterized by its affordability and possesses notable advantages in the field of tissue engineering. This is primarily attributed to its remarkable ability to effectively transport therapeutic biomolecules [138,139]. H. Mi et al. explored SiO_2_/PVA fibers for bone tissue engineering applications [140]. Self-assembly electrospinning technique was used to prepare *tetraethyl orthosilicate* (*TEOS*)/(10%, 14%, 18%) PVA to prepare 3D fibrous silica sponges. The impact of the aging process of the prepared solutions on the morphology of the fibers was examined. It was observed that with a longer aging time, fiber diameter tends to decrease due to increased solution conductivity. The increase in the PVA content (14% and 18%) increased the fiber diameter with no bead formation. The 3D fibrous sponges showed ultra-high porosity (98%) with superhydrophilicity (7500%) and had the ability to undergo deformation into various shapes, which can be beneficial for tissue regeneration involving different geometric configurations. The fibroblast cells were cultured on silica sponges which depicted the high cell viability of the samples and cell migration into the sponges. All these properties are desirable for tissue engineering applications [140]. The in vivo study on the same silica/PVA electrospun 3D sponges was reported by A. Stoica (Oprea) et al. in which the application of these structures in bone engineering applications is reinforced [141]. 

**Figure 17:**
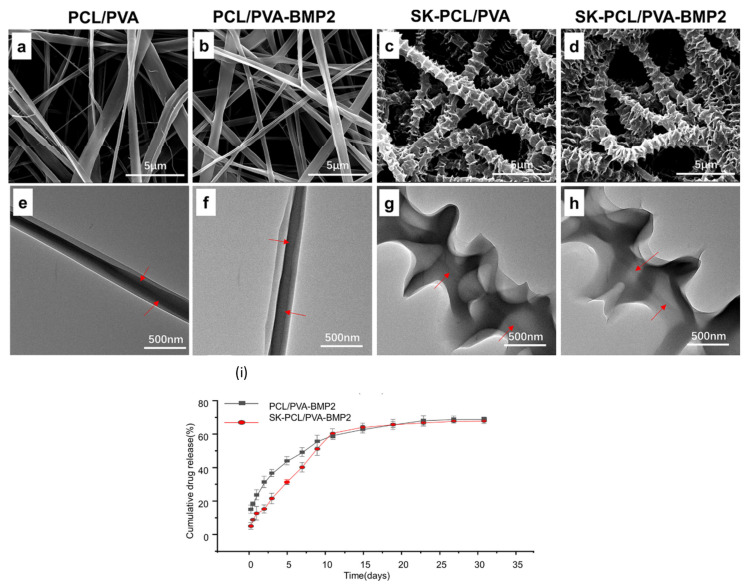
SEM micrographs of core–shell nanofiber with different formulations (**a**,**b**) and SK structures (**c**,**d**); TEM images of core–shell nanofibers with different formulations (red arrows indicate the smooth fiber) (**e**,**f**), SK structures (red arrows indicate the hierarchical “disk like” crystal lamellae formed on fibers) (**g**,**h**), and cumulative drug release profiles of nanofibers and SK structures (**i**) (reprinted with permission from ACS Publications) [151].

The coaxial electrospinning technique has also been used for the preparation of core–shell fibers for tissue engineering applications due to their significance in controlled drug release. In a recent study, core–shell fibers with PCL blended with *collagen* and hydroxyapatite (HA) as the core and, PVA blended with *oregano extract* and mesoporous silica as the shell are explored for their bone regeneration potential [142]. Bead-free and fine morphology of the composite fibers was observed. The presence of PVA enhanced the swelling ability of the fiber mats and the degradation percentage of samples was found to be 56.15% after 14 days. Moreover, the porosity of the prepared fiber mats was comparable to that of the natural bone. Cell viability assessment for fibroblast cells and MG63 cells showed no cell toxicity associated with the prepared samples. The presence of *oregano extract* was believed to be a promoting factor to enhance cell proliferation. Furthermore, the in vitro osteogenic evaluation of the fiber meshes exhibited higher stimulatory activity in cellular differentiation and enhanced the mineral deposition which indicated their improved osteogenic property. *Adenosine* (*Ade*) has its advantages in simulating bone formation, however, the systematic administration of *Ade* has severe side effects, along with its short half-life which limits its clinical applications [143,144,145]. X. Cheng et al. reported controlled drug release of PCL/PVA+ *Ade* core–shell fiber mats for bone-regeneration activity [146]. The core was comprised of 0.3:0.4 (*w*/*w*) of *Ade* and PVA while PCL polymer was used for the shell of the core–shell fibers prepared using the coaxial electrospinning technique. The uniform morphology of the fibers was observed with an average diameter of 2019 ± 1052 nm. High mechanical strength was observed for the prepared fiber mats. The drug release analysis revealed a controlled release of drug for 6 days which can be attributed to the slow degradation of PCL. The effect of *Ade* on the osteogenic differentiation of bone mesenchymal progenitor cells (BMSCs) suggested that its controlled release facilitated the process with no cytotoxicity for BMSCs. In vivo analysis revealed that the PCL/PVA membranes aided in sustained drug release of Ade to restore large bone defects without causing the side effects that are usually associated with Ade when administered systematically. Since for tissue regeneration, the fibrous scaffolds should ideally mimic the ECM, therefore, to improve the surface of the ECM, self-induced crystallization assists in attaining the hierarchical self-induced nanohybrid shish-kebab (SK) structure scaffold. SK structure is comprised of nanofiber (shish) and disc-shaped lamellae (kebabs). Such structure improves cell attachment, proliferation, and mineralization, and it has extensively been used for bone regeneration applications [147,148,149,150]. An innovative study was reported by C. Huang et al. to analyze the hierarchical core–shell fiber structure for its bone regeneration efficacy [151]. PCL was utilized as shell material and PVA incorporated with *bone morphogenetic protein 2* (*BMP2*) was the core of the fibers. The coaxial electrospinning technique was used to prepare the core–shell fibers PCL/PVA-*BMP2*. The prepared fibers were processed through the solvent-evaporation method to successfully attain shish-kebab (SK) core–shell morphology. PCL/PVA-*BMP2* and SK-PCL/PVA-*BMP2* showed fine morphology as shown in SEM images and TEM micrographs in Figure 17. Introducing SK structure in core–shell nanofibers assisted in improving the mechanical strength and controlled drug release. *BMP2* is known to enhance the proliferation and osteogenic differentiation of BMSCs which was also proven in this study. In vitro drug release showed the sustained drug release for SK-PCL/PVA-*BMP2* which depicted the significance of SK morphology in avoiding the burst release. The sustained drug release is attributed to the slow degradation of PCL due to the SK structure. Moreover, an in vivo study revealed that the SK structure assisted in bone formation with the presence of *BMP2*, and it also enhanced the BMSC attachment and proliferation. The highest bone formation efficacy of 76.38 ± 4.13% was observed for the SK-PCL/PVA-*BMP2* formulation. 

M. Rama and U. Vijayalakshmi evaluated the incorporation of silk fibroins (SFs) with PVA and HA to prepare the electrospun fiber mats for osteoregenerative characteristics [152]. PVA/HA/SF electrospun 3D scaffolds were prepared with an average fiber diameter of 1 mm and bead-free fine morphology. The scaffolds were processed through a freeze and drying procedure for solidification purposes. After 7 and 14 days of immersion in simulated body fluid (SBF) solution, the biomineralization of the scaffolds tended to increase. Moreover, the degradation of the scaffolds was observed to increase with increasing immersion duration. The samples subjected to freeze and drying procedure showed higher cell proliferation for osteoblasts cell line MG 63. In vitro drug release study of *Cephalexin Monohydrate*-loaded PVA/HA/SF (without freeze-drying) revealed that the presence of SF in the scaffolds aided in sustained drug release characteristics. All these characteristics exhibit that PVA/HA/SF (without freeze-drying) can facilitate osteoregeneration. SFs have also been used in core–shell fibers by coaxial electrospinning process for bone regeneration. G. Cheng et al. evaluated SF/PCL/PVA core–shell fibers with *BMP2* loaded core and surface coated with *connective tissue growth factor* (*CTGF*) for their potential in controlled co-delivery [153]. Layer-by-layer (LBL) surface coating of the core–shell fiber surface was performed by CS solution (positively charged) and CTGF solution (negatively charged). The core was comprised of *BMP2*/PVA and the shell was composed of (SF/PCL)_1:5_. The co-delivery of *CTGF* and *BMP2* was analyzed to study the angiogenesis and osteogenesis activity, respectively. LBL has aided the co-delivery of *CTGF* and *BMP2* promoting vessel formation and bone recovery. Transient release of *CTGF* enhanced the pro-angiogenic effect and sustained release of *BMP2* promoted the osteogenic effect for bone regeneration. Further recent studies with significant results have been tabulated in Table 3.

Electrospinning has been widely utilized in the development of drug delivery systems using polyvinyl alcohol (PVA), particularly for the purpose of tissue regeneration, specifically in the context of bone regeneration. Researchers have carefully optimized the process parameters to achieve a bead-free morphology of the fibers. Various research groups have successfully generated PVA-based 3D scaffolds with excellent swelling characteristics, which have been shown to promote tissue regeneration. Encapsulation of bone morphogenetic protein 2 (BMP2) within core–shell fibers has demonstrated promising results in promoting bone ossification without causing any cytotoxic effects. To enhance the drug release properties, cell attachment, and proliferation, innovative techniques such as surface modification and layer-by-layer surface coating of the fibers have been employed. While the presence of PVA in uniaxial and core–shell fibers has shown improvements in bone regeneration, there is a lack of studies investigating the use of PVA-based triaxial fibers in this field. 

### 3.4. Other Electrospun PVA-Based DDS

Electrospun PVA-based drug delivery systems have also been developed for cancer therapy, corneal tissue engineering, and oral drug delivery. Despite the limited number of studies conducted in the mentioned domains, the progress made in electrospinning-based drug delivery systems utilizing PVA holds the potential for significant breakthroughs in these fields.

Cancer is a lethal disease that poses challenges in treatment due to its varied symptoms. Polymeric materials have emerged as a promising field of research as they offer the potential to encapsulate, protect, transport, and administer therapeutic agents. In particular, PVA and its characteristics have been extensively studied as it is a commonly used material in pharmaceutical formulations. PVA is often combined with other polymers or materials to enhance the properties of the composite and improve its absorption. 

PVA also possesses good optical characteristics which have been utilized for corneal tissue engineering.

Some of the significant and recently reported studies are presented in Table 4.

## 4. Conclusions

This review reported on the use of electrospun drug delivery systems developed using PVA. Electrospinning has proven to be successful in a wide range of biomedical applications. Research will continue to explore its various uses due to its versatility, cost-effectiveness, and ease of use and fabrication in any research facility, even with limited financial resources. Electrospinning parameters such as the molecular weights of the polymer and drugs, solution viscosity/volatility, flow rate, applied voltage, needle gauge, and needle-to-collector distance, play a critical role in designing the desired drug delivery system. PVA has significantly been utilized for transdermal patches, wound dressings, tissue engineering, and cancer therapy, mainly due to its hydrophilicity, biocompatibility, non-toxicity, and mechanical strength. It has been mostly used in uniaxial and coaxial electrospun fibers for different drug delivery systems. Drug-loaded PVA in the core promotes the drug release characteristics and hence their therapeutic effect. When it is blended with other polymers, especially chitosan, the crosslinking of the fibers is an important factor that eventually affects the drug-release characteristics of the fiber mats. The fiber morphology and porosity play a major role in the drug release properties of electrospun drug delivery systems; therefore, the solution properties are tailored accordingly. 

### 4.1. Developments

The surface modification of electrospun fibers has also been recently studied to enhance cell attachment and proliferation. Moreover, the surface modification of the fibers leads to enhanced tissue regeneration. The innovative techniques, especially for the surface modification of the fibers, have led to improvements in the efficacy of the drug delivery systems with time. Furthermore, the utilization of innovative multilayered fibrous structures incorporating polyvinyl alcohol (PVA) has demonstrated significant advancements in promoting cell adhesion and proliferation, ultimately leading to enhanced efficacy of the corresponding drug delivery systems.

### 4.2. Challenges

Although electrospinning has been used successfully for the development of various drug delivery systems, there remain fundamental questions regarding the behavior of PVA in blends that must be addressed to ensure the diversity of multifunctional electrospun drug delivery systems and to effectively control the structural complexity and interfacial interactions for expedited treatment. These concerns necessitate the refinement of electrospinning experimental parameters, the implementation of systematic approaches, and the advancement of specific knowledge.

### 4.3. Future Outlook

Further studies are needed to fully understand the characteristics of previously created micro- and nano-scale fibers, both in vitro and in vivo. Additionally, while some nanofibrous scaffolds have been extensively studied in vitro, it is now important to focus on their interactions with native tissues in vivo. The exploration of PVA-based electrospun triaxial fibers has been limited, and further attention is required to fully utilize PVA in the development of drug delivery systems. It is well-known that multi-axial fibers have high drug loading efficiency. Incorporating PVA into triaxial fibers will not only address the challenges associated with drug loading efficiency, but can also facilitate controlled drug release if appropriately designed.

## Figures and Tables

**Table 1:** Limitations and advantages of the most commonly used natural and synthetic polymers for electrospun DDSs.

Polymer, Type of Polymer	Limitations	Advantages	Ref.
**Chitosan** ***Natural***	Extremely hydrophilic which leads to loss of nanofibrous structure, high degradation rate, and poor mechanical strength	Non-toxic, and biodegradable qualities make it biocompatible with a wide range of organs, tissues, and cells.	[9,22]
**Gelatin** ***Natural***	Rapid degradation, poor mechanical strength, and complete dissolution	Intrinsic bioactivity, high biocompatibility, cell adhesion, biodegradability, low immunogenicity	[23,24]
**Hyaluronic Acid** ***Natural***	High viscosity, high surface tension, low evaporability, high electrical conductivity that may lead to electrospinning circuit failure	Biocompatibility, non-immunogenicity, biodegradability, excellent tumor-targeting ability	[25,26,27,28]
**Collagen** ***Natural***	Variability in enzymatic degradation rate (depending on enzyme concentration), difficult to maintain its dimension in vivo due to swelling, poor mechanical strength, in vivo (not suitable for load-bearing tissues)	Biocompatible, non-antigenic, non-toxic, biodegradable (degradation can be regulated via crosslinking), compatible with synthetic polymers, promotes blood coagulation	[29,30]
**Alginate** ***Natural***	Low solubility, high viscosity due to high MW, high density of hydrogen bonding, polyelectrolyte nature of aqueous solution, and lack of appropriate organic solvent.	High water content, nontoxicity, soft consistency, biocompatibility, biodegradability, low immunogenicity	[31,32]
**PLA** ***Natural***	Poor mechanical strength, low cell adhesion because of its hydrophobicity, biological inertness, acidic degradation products, inflammation in vivo	Biocompatible, biodegradable by hydrolysis and enzymatic activity, low immunogenicity	[33,34]
**PLGA** ***Synthetic***	Poor hydrophilicity, poor cell adhesion, higher viscosity, production of acids upon degradation	Strong biodegradability, suitable for controlled-release drug delivery of medicines, peptides, proteins, and other substances	[35,36]
**PCL** ***Synthetic***	High hydrophobicity, poor bioactivity, low mechanical strength, and higher amount of PCL reduces the swelling capability of DDS	Slower degradation rate, shorter in vivo adsorbable time, generation of a minimal acidic environment during degradation	[37,38]
**PEG** ***Synthetic***	Low molecular weight makes it challenging to electrospin	Non-toxic, non-immunogenicity, good biocompatibility, and anti-protein adsorption	[39,40]

The bold and italic texts in Column 1 signify the name of the polymer and its category, respectively.

**Table 2:** Further significant studies for wound healing applications using electrospun PVA.

Formulations	Electrospinning Type and Morphology	DDS Type	Conclusive Remarks	Ref
PVA, PVA/*astragalus polysaccharide* (*APS*), PVA/*APS*/*astragaloside IV* (*ASL*)	***Uniaxial*** Fine fiber morphology with average diameters of 210.56 ± 91.30, 138.679 ± 93.616, and 145.68 ± 66.856 nm, respectively.	Diabetic wound healing in vivo	*ASL*/*APS*/PVA showed outstanding results in inhibiting inflammation, assisting collagen deposition, and better wound re-epithelialization. Large area healing (94.5 ± 6.1%), basal congestion at the center of the wound, massive tissue proliferation with no infection. PVA alone did not show a promising wound healing rate.	[118]
PVA, PVA/*Snail Mucus* (*SM*), PVA/*Ag-SM*	***Uniaxial*** Bead-free fine morphology and homogeneous fiber mats for all formulations with average diameters of 170, 126, and 110 nm, respectively.	Wound Healing In vitro/In vivo	After a sharp release for an initial 6 h, sustained drug release was observed for 72 h. Significantly high cell viability of HSF-PI 18 fibroblast cells. PVA/*Ag-SM* inhibited bacterial growth and enhanced the wound-healing process.	[119]
PVA/CS-g-Poly (N-vinyl imidazole) /TiO_2_/*CUR*	***Uniaxial*** PVA/CS-g-Poly (N-vinyl imidazole) /18.5%TiO_2_/25%*CUR* formulation showed fine fiber morphology with an average diameter of 245 ± 40 nm. PVA/CS-g-Poly (N-vinyl imidazole)/97%TiO_2_/150%*CUR* exhibited fine fiber morphology with average diameter of 319 ± 50 nm	Wound Healing In vitro/In vivo	Heated PVA/CS-g-Poly (N-vinyl imidazole) /97%TiO_2_/150%*CUR* formulation avoided burst release and slower drug release characteristics. Superior antibacterial activity against *S. aureus* (99.9% in 24 h) and *E. coli* (85% in 24 h) with no toxicity to healthy fibroblasts. PVA/CS-g-Poly (N-vinyl imidazole)/18.5%TiO_2_/25%*CUR* formulation showed good mechanical strength and complete wound healing in 14 days.	[120]
5% (*w*/*w*) *Eugenol (EUG)*-incorporated PCL/PVA/CS	***W/O and O/W Emulsion*** W/O emulsion with 5% *EUG* showed fiber morphology with high bead density. O/W emulsion with 5% *EUG* showed fewer beads with uniform fiber formation. The average diameters of fibers produced from W/O and O/W were 387.07 ± 179.51 nm and 174.47 ± 38.93 nm, respectively.	Wound healing In vitro	W/O emulsion showed better inhibiting properties against *S. aureus* (92.43%) and *P. aeruginosa* (94.68%) as compared to O/W emulsion (83.08% and 87.85%, respectively). O/W exhibited superior in vitro drug release properties.	[121]
0.0%, 1.5 and 2.5% (*w*/*v*) *Pistacia atlantica oil* (*PAO*) in PVA/sodium alginate (ALG)	***Uniaxial*** Bead-free fine fiber morphology with average diameters of 191 ± 15, 237 ± 18, and 259 ± 10 nm. Fiber diameters increase with %*PAO*	Wound healing In vitro/In vivo	Mean fiber diameter increased with *%PAO.* PVA/ALG-*1.5%* (*w/v*) *PAO* showed suitability for wound dressings due to its promising antimicrobial activity and providing moisture to the wound site while allowing oxygen exchange. In vivo study showed 92.07% wound healing.	[122]
*Bilayer fibrillar scaffold immobilized with epidermal growth factor* (*EGF*) PCL as upper layer, CS/PVA as lower layer	***Uniaxial*** Randomly aligned, bead-free, fine fibers with an average diameter of 238.36 ± 36.99 nm for the CS/PVA layer, 1271.79 ± 428.49 nm for the PCL layer	Wound healing In vitro/In vivo	In vitro analysis showed that a bilayer design immobilized with *EGF* possessed suitable biological properties for wound dressing applications. A 14-day In vitro analysis confirmed that EGF immobilized scaffold promoted wound healing, similar to commercial wound dressing.	[123]
*40%* (*w/w*) *Ipomoea pes-caprae* (*IPC*) *leaf-extract*-loaded PVA (10% (*w*/*w*))	***Uniaxial*** Homogeneous and smooth fiber morphology with an average diameter of 100 nm.	Wound healing In vitro	Electrospun hydrogel showed swelling 102 ± 7.45% as compared to conventional hydrogel 68.60 ± 6.72% which is attributed to the greater surface area of electrospun hydrogels. Higher drug loading capacity was observed for electrospun hydrogels. *IPC* leaf-extract-loaded electrospun hydrogels demonstrated desirable antimicrobial activity against *S. aureus.*	[124]
*Lysine* (*Lys*)-loaded PVA *IBP-Lys* -loaded PVA *Lavender oil* (*LO*)*-Lys*-loaded PVA	***Uniaxial*** Bead-free uniform fiber morphology was observed for all samples. Average diameters were 474.22 ± 144.85 nm, 385.03 ± 108.21 nm, and 487.14 ± 155.81 nm, respectively.	Skin Regeneration *Antimicrobial*	All the electrospun membranes presented suitable morphological, mechanical, physiochemical, and biological properties to be used as wound dressings. The *LO* incorporation on PVA_*Lys* membranes mediated a strong antibacterial effect against both *S. aureus* and *P. aeruginosa.*	[125]

Italic text in column 1, 2 and 3 represents the drugs/bioactive agents incorporated in fibers. Bold/Italic text in column 2 signifies the type of Electrospinning technique used for each study.

**Table 3:** Further studies highlighting the potential of electrospun PVA for tissue engineering.

Formulations	Electrospinning Type and Morphology	DDS type	Conclusive Remarks	Ref
*Platelet-rich plasma* (*PRP*)-incorporated SF/PCL/PVA *PRP*:PVA (10:0, 9:1, 8:2, and 7:3) for the core	***Coaxial*** SF/PCL/ (PRP-PVA)_7:3_ nanofibrous scaffolds showed uniform morphology among all formulations, with an average diameter of 385.9 ± 84.6 nm.	Bone Tissue Engineering In vitro/In vivo	The *PRP*-derived growth factors, released from the SF/PCL/*(PRP*-PVA)_7:3_ scaffolds, exhibited sustained release for nearly 30 days and positively influenced the proliferation, migration, and osteogenesis of BMSCs in vitro and in vivo.	[154]
*Tri-layer fibers* Layer I: PCL Layer II: PCL/Cellulose acetate (CA)-loaded with *5 wt% beta-tri calcium phosphate* (*β-tcp*) Layer III: PVA/Poly(vinyl Acetate) (PVAc)-loaded with *5 wt% simvastatin* (*SIM*)	***Uniaxial*** Fine fiber morphology was observed for all layers with average diameters of 736.052 nm (Layer I), 668.28 nm (Layer II), and 281.14 nm (Layer III).	Bone Tissue Regeneration In vitro	The fabricated ECM mimicking composite nanofibers loaded with *β-tcp*, *SIM* shows excellent bioactivity inducing precipitation of bone-like apatite minerals on its surface under simulated physiological conditions. In vitro cell culture test revealed that the incorporation of *β-tcp* and *SIM* into the composite nanofiber enhanced osteoblast cell adhesion and proliferation than the control fiber. The characteristics depicted the potential of the Tri-layered fibrous structures in bone regeneration.	[155]
*Doxorubicin* (*DOX*)-loaded PVA/poly(butylene carbonate)(PBC)	***Coaxial*** ***DOX + PVA as core, PBC as shell*** Lower and equal feed rate ratios for PVA/PBC (1:1.3, 1:1) showed non-uniformity in fiber diameter. Fine morphology was observed for feed rate 1:0.7 with an average diameter of 42 nm.	Chemotherapy/Tissue engineering In vitro	In vitro analysis revealed that *DOX*-loaded core–shell PVA/PBC nanofibers were effective in prohibiting SKOV3 ovary cell attachment and proliferation. The prepared fibers were degraded in a physiological environment	[156]
*Eumelanin nanoparticles* (*EUNp*)/PVA	***Uniaxial*** Bead-free fine fiber morphology was observed with an average fiber diameter of 161.40 ± 8.86 nm.	Skeletal Muscle Tissue Engineering In vitro	*EUNp*/PVA nanofibrous scaffolds exhibit inherent physiochemical characteristics along with high electrical conductivity and structural integrity. The composites promoted guided reorganization of C2C12 myoblasts towards myotube-like structure formation within a week.	[157]
PVA, *Bioactive glass* (*BG*)-coated PVA scaffolds	***Uniaxial*** Bead-free and homogeneous morphology for PVA was observed with an average diameter of 286 ± 14 nm. For *BG-*coated PVA scaffolds homogeneity of the fibers was reduced, however, no bead formation was observed. The average diameter was 318 ± 36 nm.	Bone Regeneration In vitro	*BG-*coated PVA scaffolds revealed superior mechanical properties as compared to PVA fibers. In vitro, the BG-coated PVA scaffolds showed a better capacity to support the proliferation of osteogenic MC3T3-E1 cells, ALP activity, and mineralization.	[158]

Italic text in column 1, 2 and 3 represents the drugs/bioactive agents incorporated in fibers. Bold/Italic text in column 2 signifies the type of Electrospinning technique used for each study.

**Table 4:** Research studies electrospun PVA for cancer therapy oral drug delivery and ocular drug delivery.

Formulations	Electrospinning Type and Morphology	DDS Type	Conclusive Remarks	Ref
***Gold nanoparticle* (*AuNP*)**-loaded PVA *CUR*-loaded PCL	***Uniaxial*** Uniform bead-free fiber morphology for both formulations was observed with diameters in the range of 300 nm, and 600–800 nm, respectively.	Skin Cancer In vitro	The anticancer activity on skin cancer cell lines by the preliminary in vitro assay of the drug was confirmed. The cell line studies revealed that the treatment of nanofibers in cancer cells exhibited more cytotoxicity than in the normal cells where similar concentrations were used thereby proving selective toxicity.	[159]
***Doxorubicin hydrochloride* (*DOX*)**-loaded Polyhydroxyalkanoate (PHA)/PVA	***Uniaxial for DOX-loaded PVA*** **(*1:100*) *fibers.*** ***Spin coating for PHA on DOX-loaded PVA fibers to obtain porous membrane.***	Chemotherapy for Colon cancer In vitro	The composite membranes had an outstanding pH sensitivity for the *DOX* release, which was desirable for clinical applications. The Caco-2 cells were almost apoptotic after being cultured for 6 days. The results suggested a high potential of the prepared membranes treating colonic carcinoma.	[160]
Tri layered nanofibers Layer I: *5-fluorouracil* (*5-FU*)loaded PCL Layer II: 5-FU-loaded PVA/methyl cellulose(MC) Layer III: *5-FU*-loaded PCL	***Uniaxial*** Bead-free fiber formation was observed for drug-loaded layers with an average diameter of 258.6 nm	Skin Cancer In vitro	Controlled drug release was obtained by incorporating *5-FU* into multilayered nanofibers. The prepared formulation revealed regulated drug release’s greater capacity to prevent negative side effects, which is a characteristic of anticancer drugs. Moreover, the multilayer structure allows for straightforward but efficient dosage modifications.	[161]
***AuNPs/paclitaxel* (*PTX*)*/camptothecin* (*CMPT*)**-loaded PVA/k-carrageenan/pegylated-PU composite fibers ***AuNPs/paclitaxel* (*PTX*)*/camptothecin* (*CMPT*)**-loaded PVA/k-carrageenan/pegylated-PU core–shell fibers	***Uniaxial for composite fibers*** ***Coaxial for core–shell fibers*** The bead-free morphology was observed with a mean fiber diameter of 225 nm at optimized process parameters for composite fibers. The increase in shell feed rate (0.3 mL/h to 0.7 mL/h) increases the fiber diameter from 330 nm to 640 nm at 20 kV.	Lung Cancer In vitro/In vivo	The higher DEE (drug entrapment efficiency) than 95% for PTX and CMPT confirmed an effective loading of anticancer drugs into the nanofibers. The maximum cytotoxicity was 75% in the presence of PVA/k-carrageenan/CMPT/Au/pegylated-PU/PTX core–shell nanofiber. In vivo release studies indicated that in rats fed with core–shell nanofibers, the blood concentration of CMPT and PTX reached the highest values of 26.8 ± 0.04 µg/mL and 26.5 ± 0.05 µg/mL in 36 h and 24 h and kept in the constant values between 36 and 84 h, and 24 and 48 and finally reduced after 84 h and 48 h, respectively. In vivo antitumor efficacy results of A549 tumor-bearing mice treated with composite and core–shell nanofibers demonstrated the best effect on the reduction in tumor volume and enhancement in tumor inhibition	[162]
***Doxorubicin* (*DOX*)**-loaded N-carboxymethyl CS (N-CMCS)-PVA/PCL composite and core–shell fibers	***Uniaxial for composite fibers*** ***Coaxial for core–shell fibers*** Beaded fiber morphology was observed for composite fibers. Bead-free morphology was observed for core–shell fibers with an average diameter of 410 nm.	Breast Cancer In vitro	DEE for core–shell nanofibers was found to be higher than composite fibers. Initial burst release of DOX was observed for composite for 11 days and 3 days, and physiological and acidic pH. Sustained drug release for core–shell fibers without initial burst release for 20 days and 10 days at pH 5.5 and 7.4, respectively. Core–shell nanofibers exhibited higher drug encapsulation efficiency, sustained release of DOX, lower adsorption capacity, higher cytotoxicity of MCF-7 breast cancer, and high biocompatibility.	[163]
(i) Non-aligned fibers; 7% collagen (COL), 10%PVA, 7%PVA/Collagen (COL), 9% PVA/Collagen (COL) (ii) Well-aligned fibers; 7% Collagen (COL), 10%PVA, 7%PVA/COL, 9% PVA/COL	***Uniaxial*** For non-aligned fibers, average diameters were 301.5 ± 74.2, 302 ± 37.9, 211.6± 142.5, and 262.9 ± 199.3 nm For aligned fibers, average diameters were 431.8 ± 72.6,204 ± 55, 183.3 ± 96.7, and 163.1 ± 103.2 nm. Fine morphology for both non-aligned and aligned fibers was observed	Corneal Tissue Engineering In vitro	Human keratocytes (HKs) and human corneal epithelial cells (HCECs) exhibited good adhesion and proliferation when cultured on electrospun scaffolds made of aligned and random PVA/COL nanofibers. The aligned nanofibers promoted organized growth in HKs, suggesting that the designed PVA/COL composite nanofibrous electrospun scaffold holds promise for use in tissue-engineered cornea.	[164]
8 combinations of ***Pramipexole*** **(*Prami*)**-loaded PVA/carboxymethylcellulose (CMC) + PCL hybrid fibers GA crosslinking was performed	***Uniaxial* (*Co-electrospinning*)** Fibers with a diameter smaller than 500 nm for PVA/CMC/Prami fibers, and a diameter larger than 500 nm for PCL nanofibers; the average diameter of fibers in this hybrid nanofibers is 931 ± 618 nm	Oral drug delivery for Parkinson’s Disease In vitro	Nanofiber PCL/PVA/CMC, which underwent a 12-h exposure to GA vapors, exhibited the most favorable release profile among the eight nanofibers tested. This particular formulation demonstrated the longest duration of drug release during the initial 8-h period, while also exhibiting an acceptable level of cytotoxicity. The nanofibers can be concentrated into a new capsule formulation to decrease the amounts of additives used in tablet form and simplify the manufacturing process.	[165]
*5%(w/w) Melatonin* (*MLT*)-loaded (5%, 6%, **7 wt% (w/v) PVA/Polyethylene oxide (PEO)**	***Uniaxial*** Bead-free fine morphology of drug-loaded fibers (7 wt%) was observed with a diameter in the range of 300–700 nm	Oral Drug Delivery In vitro	The electrospun blend PVA/PEO fibers loaded with MLT showed great compatibility with human umbilical vein endothelial cells (HUVECs) for a period of 24 h, indicating their potential application in topical and/or mucoadhesive drug delivery.	[166]

Italic text in column 1, 2 and 3 represents the drugs/bioactive agents incorporated in fibers. Bold/Italic text in column 2 signifies the type of Electrospinning technique used for each study.

## Data Availability

Not applicable.
